# Tuning Enzyme Thermostability via Computationally Guided Covalent Stapling and Structural Basis of Enhanced Stabilization

**DOI:** 10.1021/acs.biochem.2c00033

**Published:** 2022-05-25

**Authors:** Jacob A. Iannuzzelli, John-Paul Bacik, Eric J. Moore, Zhuofan Shen, Ellen M. Irving, David A. Vargas, Sagar D. Khare, Nozomi Ando, Rudi Fasan

**Affiliations:** † Department of Chemistry, University of Rochester, Rochester, New York 14627, United States; ‡ Department of Chemistry and Chemical Biology, Cornell University, Ithaca, New York 14853, United States; § Department of Chemistry and Chemical Biology, Rutgers University, Piscataway, New Jersey 08854, United States

## Abstract

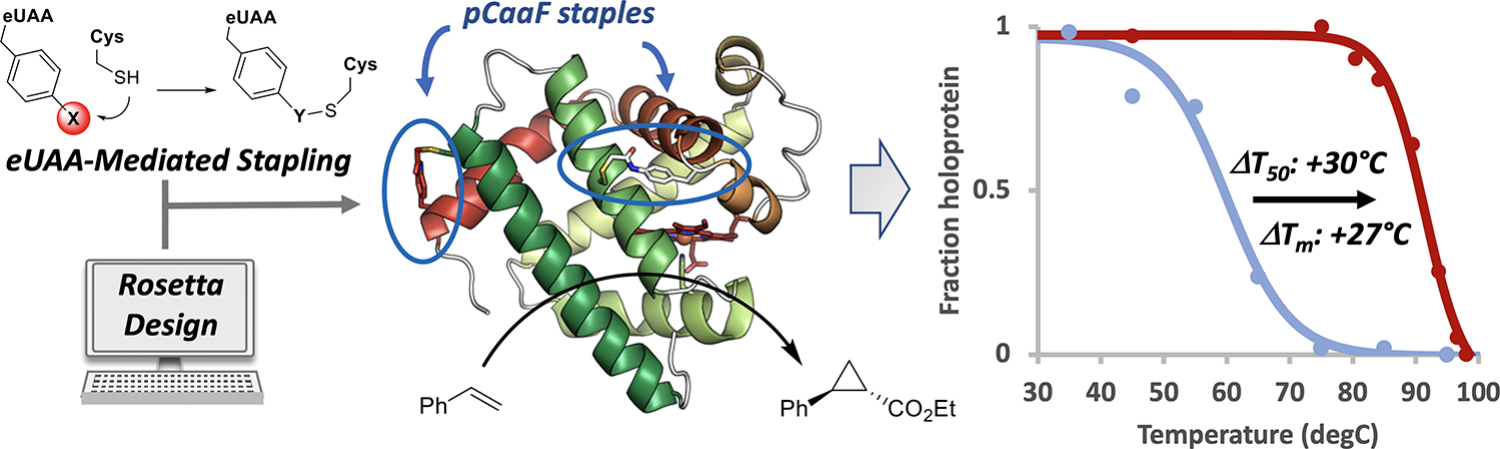

Enhancing the thermostability of enzymes without impacting their catalytic function represents an important yet challenging goal in protein engineering and biocatalysis. We recently introduced a novel method for enzyme thermostabilization that relies on the computationally guided installation of genetically encoded thioether “staples” into a protein via cysteine alkylation with the noncanonical amino acid *O*-2-bromoethyl tyrosine (O2beY). Here, we demonstrate the functionality of an expanded set of electrophilic amino acids featuring chloroacetamido, acrylamido, and vinylsulfonamido side-chain groups for protein stapling using this strategy. Using a myoglobin-based cyclopropanase as a model enzyme, our studies show that covalent stapling with *p*-chloroacetamido-phenylalanine (pCaaF) provides higher stapling efficiency and enhanced stability (thermodynamic and kinetic) compared to the other stapled variants and the parent protein. Interestingly, molecular simulations of conformational flexibility of the cross-links show that the pCaaF staple allows fewer energetically feasible conformers than the other staples, and this property may be a broader indicator of stability enhancement. Using this strategy, pCaaF-stapled variants with significantly enhanced stability against thermal denaturation (Δ*T*_m_′ = +27 °C) and temperature-induced heme loss (Δ*T*_50_ = +30 °C) were obtained while maintaining high levels of catalytic activity and stereoselectivity. Crystallographic analyses of singly and doubly stapled variants provide key insights into the structural basis for stabilization, which includes both direct interactions of the staples with protein residues and indirect interactions through adjacent residues involved in heme binding. This work expands the toolbox of protein stapling strategies available for protein stabilization.

## Introduction

The thermostabilization of enzymes represents an important and long-standing goal in protein engineering as it greatly expands the reaction conditions available for biocatalysis and other biotechnological applications.^1−5^ In the context of industrial biocatalysis, for example, thermostable enzymes can offer longer half-lives, increased robustness to lyophilization, and robustness to elevated reaction temperatures, which can improve catalytic rates and substrate solubility.^6−8^ In addition, increased enzyme thermostability is often accompanied by improved stability in the presence of organic cosolvent(s), high substrate (or product) concentrations, and/or other denaturing agents and reaction conditions.^9−12^ Thermostabilization is also beneficial toward increasing the robustness of proteins and enzymes to mutagenesis and protein engineering, facilitating the acquisition of novel or improved functional properties during directed evolution.^13,14^

Over the past two decades, several protein engineering strategies have been investigated for enhancing the thermostability of enzymes, including directed evolution using random,^15,16^ structure-guided,^17,18^ or global mutagenesis^19^ as well as bioinformatic approaches based on consensus mutagenesis^9,20−23^ and ancestral sequence reconstruction.^24−26^ Rational design strategies have included the introduction of intramolecular disulfide bridges,^27−30^ targeted mutagenesis to flexible regions within the protein,^31,32^ and computational design methods.^33−42^ Despite this progress, identifying beneficial mutations that stabilize an enzyme scaffold without sacrificing catalytic activity and/or without the requirement of extensive screening efforts has remained a considerable challenge. Protein stabilization by means of genetically encoded amino acids or chemical cross-linkers was also investigated.^29,43−46^

**Figure 1:**
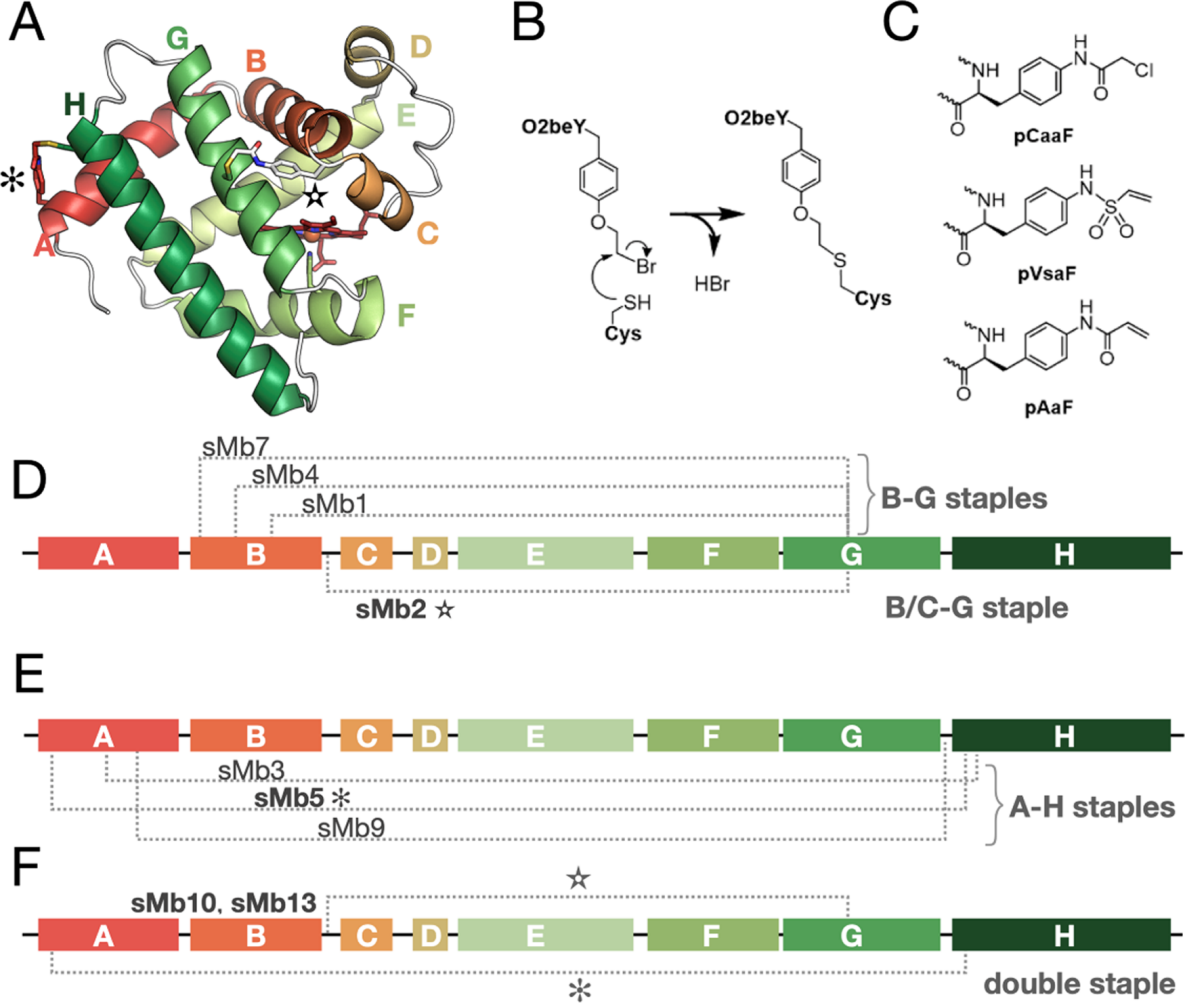
Design of stapled myoglobin constructs. (A) Structure of the doubly stapled sMb13 mutant determined in this study with the myoglobin helices labeled A–H. Locations of the staples in sMb2 (☆) and sMb5 (*) are indicated. (B) The scheme depicts the stapling reaction between the nonproteinogenic amino acid *O*-2-bromoethyl tyrosine (O2beY) and cysteine forming a redox-stable thioether bond. (C) Structures of alternative electrophilic unnatural amino acids (eUAAs) investigated in this study. (D) Topology of the mutants with stapling between helix B and helix G, compared with that of sMb2, which contains a staple between the B/C loop and helix G. (E) Topology of the mutants with stapling between the N-terminal helix A and the C-terminal helix H. (F) Topology of the doubly stapled sMb10 and sMb13 mutants includes sMb2 and sMb5 staples. sMb13 additionally includes a charge-compensating H113E mutation.

Recently, we introduced a novel strategy for enzyme thermostabilization that relies on the post-translational introduction of thioether “staples” formed via an S_N_2 reaction between a cysteine residue and the genetically encodable, noncanonical amino acid *O*-2-bromoethyl tyrosine (O2beY) (Figure 1A,B).^47^ In this method, computational design using the Rosetta framework is employed to determine the optimal positions for the introduction of intramolecular cross-links within the target protein scaffold. Using a myoglobin-based cyclopropanase as the model enzyme, this approach enabled the identification of two beneficial stapling sites, which when combined led to a doubly stapled variant with significantly increased thermostability compared to the parent enzyme (Δ*T*_m_′ = +18 °C; Δ*T*_50_ = +16 °C, where *T*_m_′ is measured as a function of the protein secondary structure and *T*_50_ is a function of heme binding). Building upon this work, we investigated here the feasibility of extending this approach to a broader range of electrophilic, cysteine-reactive noncanonical amino acids (eUAAs) featuring chloroacetamido, acrylamido, and vinylsulfonamido side-chain groups for protein stapling (Figure 1C). The present study demonstrates the functionality of these eUAAs for protein stapling and reports the identification of a cysteine-reactive eUAA, namely, *p*-chloroacetamido phenylalanine (pCaaF), capable of offering superior stapling efficiency and stapling-induced thermostabilization in a myoglobin-based biocatalyst, compared to the previously reported O2beY-based strategy. In addition, using a combination of crystallographic analyses and molecular dynamics simulations, we have obtained first-time insights into the structural basis of protein thermostability enhancement through covalent stapling in this system.

## Experimental Details

### Materials and Reagents

All of the eUAAs used for protein stapling were synthesized as described previously.^48,49^ Reagents for the cyclopropanation reaction (styrene, ethyl diazoacetate (EDA)) were purchased from Sigma-Aldrich.

### Molecular Cloning and Plasmids

The preparation of pEVOL-based plasmids for the expression of the orthogonal AARS/tRNA pairs for amber stop codon suppression with O2beY, pCaaF, pAaF, or pVsaF was reported previously.^48,49^ pET22-based plasmids for the expression of the sMb constructs reported in Tables 1 and 2 in His-tagged form were described previously.^47^ sMb constructs lacking a polyhistidine tag were prepared by introducing a stop codon at the end of the myoglobin sequence using the forward primer 5′-GAATTGTGAGCGGATAACAATTCC-3′ and reverse primer 5′-GTGCTCGAGCTAACCACCC-3′. The primers were used in a first polymerase chain reaction (PCR) to amplify the gene encoding for the Mb construct (∼500 bp). In a second PCR reaction, a QuikChange protocol was utilized to amplify the entire pET22 plasmid using the PCR product from the first reaction as the primer. The PCR product was incubated with *Dpn* I (10 units) at 37 °C for 1 h to remove the template plasmid. The amplified plasmid was transformed into DH5α *Escherichia coli* cells and confirmed by sequencing.

**Table 1:** Thermodynamic and Kinetic Stabilities of sMb2 and sMb5 Variants Containing Different eUAA-Based Staples^a^

entry	variant	mutations	location staple^b^	*T*_m_′ (°C) (Δ*T*_m_′)^c^	*T*_50_ (°C) (Δ*T*_50_)^d^
1	Mb(H64V,V68A)			63.0 ± 1.5 (0.0)	61.1 ± 1.7 (0.0)
2	sMb2(O2beY)	H36(O2beY), E109C	B/C–G	73.2 ± 0.2 (+10.2)	63.8 ± 0.3 (+2.7)
3	sMb2(pAaF)	H36(pAaF), E109C	B/C–G	72.9 ± 0.5 (+9.1)	62.7 ± 1.4 (+1.6)
4	sMb2(pVsaF)	H36(pVsaF), E109C	B/C–G	68.7 ± 0.5 (+5.7)	66.5 ± 3.1 (+5.4)
5	sMb2(pCaaF)	H36(pCaaF), E109C	B/C–G	79.4 ± 0.8 (+16.4)	74.6 ± 0.7 (+13.5)
6	sMb5(O2beY)	G5(O2beY), D126C	A–H	76.0 ± 2.0 (+13.0)	71.5 ± 0.9 (+10.4)
7	sMb5(pAaF)	G5(pAaF), D126C	A–H	68.4 ± 0.4 (+5.4)	69.8 ± 1.8 (+8.7)
8	sMb5(pVsaF)	G5(pVsaF), D126C	A–H	72.9 ± 0.5 (+9.9)	69.5 ± 1.2 (+8.4)
9	sMb5(pCaaF)	G5(pCaaF), D126C	A–H	79.2 ± 0.5 (+16.2)	76.9 ± 1.0 (+15.8)

a Mean values are from *n* ≥ 2 with error bars at 1 s.d.

b Corresponding to alpha helices A through H, as labeled in Figure 1A,B.

c Apparent melting temperatures as determined via circular dichroism (see Figure S1).

d Half-maximal denaturation temperature of holoprotein (Soret band) after 10 min incubation at varying temperatures.

**Table 2:** Thermodynamic and Kinetic Stabilities of the pCaaF-Containing sMb Variants vs O2beY-Containing Counterparts^a^

				*pCaaF*		*O2beY*	
entry	variant	mutations	location staple^b^	*T*_m_′ (°C) (Δ*T*_m_′)^c^	*T*_50_ (°C) (Δ*T*_50_)^d^	*T*_m_′ (°C) (Δ*T*_m_′)^e^	*T*_50_ (°C) (Δ*T*_50_)^e^
1	Mb(H64V, V68A)		n.a.	63.0 ± 1.5 (0.0)	61.1 ± 1.7 (0.0)	63.0 ± 1.5 (0.0)	61.1 ± 1.7 (0.0)
2	sMb1(UAA)	R31(UAA), S35K, E109C	B–G	71.3 ± 0.5 (+8.3)	69.5 ± 2.5 (+8.4)	67.5 ± 1.3 (+4.5)	59.1 ± 0.6 (−2.0)
3	sMb2(UAA)	H36(UAA), E109C	B/C–G	79.4 ± 0.8 (+16.4)	74.6 ± 0.7 (+13.5)	73.2 ± 0.2 (+10.2)	63.8 ± 0.3 (+2.7)
4	sMb3(UAA)	L9R, H12C, D122T, A127(UAA)	A–H	74.4 ± 0.5 (+11.4)	69.8 ± 2.2 (+8.7)	72.2 ± 1.4 (+9.2)	60 ± 3 (−1.1)
5	sMb4(UAA)	D27(UAA), H113C, V114G	B–G	64.1 ± 1.3 (+1.1)	66.5 ± 0.6 (+5.4)	56.1 ± 1.1 (−6.9)	50 ± 2 (−11.1)
6	sMb5(UAA)	G5(UAA), D126C	A–H	79.2 ± 0.5 (+16.2)	76.9 ± 1.0 (+15.8)	76.0 ± 2.0 (+13.0)	71.5 ± 0.9 (+10.4)
7	sMb7(UAA)	D20S, G23C, D27A, R118(UAA)	B–G	65.0 ± 0.6 (+2.0)	60.3 ± 0.4 (−0.8)	69.9 ± 1.4 (+6.9)	54 ± 1 (−7.1)
8	sMb9(UAA)	K16C, H119A, G121(UAA), D122S	A–G/H	70.2 ± 0.5 (+7.2)	67.0 ± 0.2 (+5.9)	64.3 ± 1.3 (+1.3)	52 ± 2 (−10.3)
9	sMb10(UAA)	G5(UAA), H36(UAA), E109C, D126C	B/C–G + A–H	89.2 ± 0.8 (+26.2)	85.6 ± 0.5 (+24.5)	82.8 ± 0.8 (+19.8)	73 ± 2 (+11.9)
10	sMb13(UAA)	G5(UAA), H36(UAA), E109C, H113E, D126C	B/C–G + A–H	90 ± 1 (+27)	91 ± 1 (+30)	84.0 ± 0.2 (+21.0)	78.3 ± 0.8 (+17.2)

a The mutations and location of the staple(s) are also indicated. Mean values are from *n* ≥ 2 with error bars at 1 s.d.

b Corresponding to alpha helices A through H, as labeled in Figure 1.

c Apparent melting temperatures as determined via circular dichroism (Figure S2).

d Half-maximal denaturation temperature of holoprotein (Soret band) after 10 min incubation.

e As previously reported in Moore et al.^47^

### Protein Expression

The pET22-based plasmid encoding the Mb variant was cotransformed into BL21(DE3) cells along with a pEVOL-based vector encoding the orthogonal AARS/tRNA pair necessary for eUAA incorporation. Recombinant cells were selected on LB agar plates supplemented with ampicillin (100 mg L^–1^) and chloramphenicol (34 mg L^–1^). Cell colonies were allowed to grow overnight at 37 °C. Colonies were used to start overnight cultures containing 5 mL of LB media supplemented with ampicillin (100 mg L^–1^) and chloramphenicol (34 mg L^–1^). Overnight cultures were used to inoculate 1 L of M9 minimal medium containing 0.5% (w/v) yeast extract, 1% (v/v) glycerol, ampicillin (100 mg L^–1^), and chloramphenicol (34 mg L^–1^). Cell cultures were grown at 37 °C until OD_600_ reached 0.5–0.6. The culture was centrifuged at 4000*g*, and the supernatant was removed. The pelleted cells were resuspended in 200 mL of M9 media containing 0.5% (w/v) yeast extract, 1% (v/v) glycerol, ampicillin (100 mg L^–1^), and chloramphenicol (34 mg L^–1^) and supplemented with l-arabinose (0.06%) and eUAA (2 mM) to induce the production of eUAA-charged tRNA. The culture was incubated for 1 h at 27 °C. Myoglobin expression was induced with the addition of IPTG (0.5 mM) and δ-aminolevulinic acid (50 mg/L), and the culture was incubated overnight at 27 °C with shaking. Cultures were harvested at 4000*g*, and the cell pellets were stored at −80 °C.

### Protein Purification

His-tagged proteins were purified via Ni-affinity chromatography, as described previously.^47^ Initial experiments with His-tagged forms of sMb2 and sMb5 variants containing pCaaF, pAaF, or pVsaF were found to give rise to varying amounts of dimers and oligomers after Ni-affinity purification. In contrast, no or minimal oligomerization was observed for the same constructs lacking the His-tag, which were thus used for the rest of the study, including crystallization. For purification, the cell pellets were resuspended in cation-exchange loading buffer (20 mM KPi, 10 mM NaCl, pH 6.0) and cells were lysed via sonication. After clarification by centrifugation, the cell lysate was loaded on a cation-exchange column (SP Sepharose Fast Flow; GE Healthcare Biosciences) and the protein was eluted with a 0 → 100% linear gradient of cation-exchange elution buffer (20 mM KPi, 500 mM NaCl, pH 6.0) for 60 min at a flow rate of 1 mL/min using a BioRad DuoFlow FPLC. Protein fractions were pooled, concentrated using an Amicon spin filter (10 KDa cutoff), and incubated overnight at pH ∼ 8.5. Protein samples were then buffer-exchanged in 50 mM potassium phosphate (KPi) buffer (pH 7.0) using an Amicon spin filter (10 KDa cutoff). For crystallization, proteins were further purified via gel filtration chromatography using a Superdex 75 10/300 GL column and isocratic elution in 50 mM KPi buffer (pH 7.0) at a flow rate of 1.0 mL/min. Protein samples for crystallization trials were buffer-exchanged with 20 mM tris buffer (pH 8.4) containing 1 mM ethylenediamine tetraacetic acid (EDTA). Protein concentration was determined based on Soret band absorption using ε_409_ = 156,000 M^–1^ cm^–1^.

### Protein Characterization

Mass spectrometry analyses were carried out using a Shimadzu performance matrix-assisted laser desorption ionization-time-of-flight mass spectrometry (MALDI-TOF MS)/MS spectrometer and sinapinic acid as matrix. Near-UV circular dichroism spectra (250–190 nm) were obtained using 3 μM solutions of purified Mb variant in 50 mM KPi buffer (pH 7.0) and recorded at 20 °C at a scan rate of 50 nm/min with a bandwidth of 1 nm and an averaging time of 10 seconds per measurement. The raw signal (θ_d_, mDeg) was background-subtracted against buffer and converted to molar residue ellipticity (θ_MRE_) using θ_MRE_ = θ_d_/(*c*  ln_R_), where *c* is the concentration (M), *l* is the path length (1 mm), and *n*_R_ is the number of residues in the protein.

### *T*_m_′ Determination

Thermal denaturation experiments were carried out using a JASCO J-1100 CD spectrophotometer equipped with variable temperature/wavelength denaturation analysis software. Samples of purified Mb variant were prepared as 3 μM solutions in 50 mM KPi buffer (pH 7.0). Thermal denaturation curves were measured by monitoring the change in molar ellipticity at 222 nm (θ_222_) over a temperature range from 20 to 100 °C (30–130 °C for constructs containing two staples). The samples were loaded into a JASCO quartz 1 mm cuvette with a capped top for analysis at 100 °C and above. The temperature increase was set to 1.0 °C per minute with a data collection interval set to 0.1 °C. Data integration time for the melt curve was set to 4 s with a bandwidth of 1 nm. Linear baselines for the folded (θ_f_) and unfolded states (θ_u_) were generated using the low-temperature (θ_f_*= m*_f_*T + b*_f_) and high-temperature (θ_u_*= m*_u_*T + b*_u_) equations fitted to the experimental data before and after global unfolding, respectively. Using these equations, the melt data were converted to a fraction of the folded protein (*F*_f_) vs temperature plots and the resulting curve was fitted to a sigmoidal equation (θ_fit_) via nonlinear regression analysis in SigmaPlot (Figures S1 and S2), from which apparent melting temperatures were derived. The reported mean values and standard errors were derived from experiments performed at least in duplicate. For a few constructs (i.e., sMb2(pAaaF), sMb5(pCaaF), sMb10(pCaaF)), small deviations between the fitting curves and experimental data were observed outside of the *T*_m_ region, which could derive from the fact that the folding process for these variants is more complex than that of a two-state unfolding model.

### *T*_50_ Analysis

For the thermal stability experiments (*T*_50_ determination), 500 μL of a 3.5 μM protein solution in 50 mM KPi buffer (pH 7.0) was incubated for 10 min at varying temperatures between 20 and 95 °C (10 °C intervals) in a thermoblock. After incubation, the protein samples were centrifuged (14,000 rpm, 4 °C, 10 min) and the supernatant was transferred to a 96-well plate. Visible spectra were recorded between 300 and 500 nm using a Tecan X microtiter plate reader. The residual fraction of holoprotein in each sample was determined based on the intensity of the Soret band (410 nm) after normalization to the sample incubated at 20 °C. Half-maximal denaturation temperatures (*T*_50_) were calculated from the fraction of the folded protein vs temperature plots by fitting the data to a four-parameter sigmoidal equation in SigmaPlot. The reported mean values and standard errors were derived from experiments performed at least in duplicate.

### Cyclopropanation Reactions

Cyclopropanation reactions were carried out at a 400 μL scale using 10 μM myoglobin, 10 mM styrene, 20 mM ethyl α-diazoacetate (EDA), and 10 mM sodium dithionite in a Coy anaerobic box. Inside the chamber, a solution of sodium dithionite (40 μL, 100 mM stock) was added to the reaction vessel containing a buffered solution (KPi 50 mM, pH = 7) of Mb. Then, styrene was added to the vessel (20 μL, 200 mM stock) followed by EDA (20 μL, 400 mM) producing a 400 μL reaction. The reaction was left under magnetic agitation for 5 h. Outside the chamber, the reactions were analyzed by adding 20 μL of internal standard (benzodioxole, 100 mM in ethanol) to the reaction mixture and extracted with 400 μL of dichloromethane and analyzed by chiral gas chromatography, as described.^47^ Yields and number of turnovers (TON) were calculated based on the amounts of cyclopropane product as determined using a calibration curve with authentic standards. All measurements were performed at least in duplicate. Diastereomeric and enantiomeric excess were determined based on the relative distribution of the four stereoisomer products, as described previously.^47^ Enantiomer resolution for compound 3b was performed by GC-FID using a Shimadzu GC-2010 gas chromatograph equipped with an FID detector and a CyclosilB column (30 m × 0.25 mm × 0.25 μm film). Separation method: 1 μL injection, injector temperature: 250 °C, detector temperature: 300 °C, column temperature set at 120 °C for 3 min, then to 140 °C at 0.85 °C/Min, and then to 245 °C at 30 °C/Min. The total run time was 31.03 min.

### Protein Crystallization and Crystallographic Analyses

For all stapled variants, 160 nL of protein in 20 mM tris pH 8.4, 1 mM EDTA, was mixed with an equal volume of reservoir solution to perform sitting-drop vapor diffusion crystallization over a 40 μL well. The protein concentrations used for crystallization were 1 mM sMb5(O2beY), 3.25 mM sMb5(pCaaF), 2.15 mM sMb13(pCaaF) (*P*2_1_2_1_2_1_), and 0.6 mM sMb13(pCaaF) (*P*2_1_) and reservoir solutions of (i) 3.0 M ammonium sulfate, 10% w/v glycerol; (ii) 2.2 M ammonium sulfate, 0.2 M sodium nitrate; (iii) 1.6 M ammonium sulfate, 0.5 M lithium chloride; and (iv) 2.2 M ammonium sulfate, 0.2 M potassium fluoride, respectively. sMb5(O2beY) crystals were cryoprotected by adding 0.6 μL of cryosolution containing 2.8 M ammonium sulfate and 20% glycerol directly to the drop. sMb5 and sMb13 (pCaaF) crystals were cryoprotected by mixing 0.6 μL of well solution and 0.6 μL of cryoprotectant solution containing 18% sucrose (w/v), 4% glucose (w/v), 16% glycerol (v/v), and 16% ethylene glycol (v/v) added directly to the crystallization drop. sMb5(O2beY) data were collected at Princeton University using a Rigaku MicroMax-007 HF rotating anode X-ray generator and a Dectris Pilatus3 R 300K detector. Data were integrated, scaled, and merged using HKL2000,^50^ and molecular replacement (MR) was performed using PHASER^51^ using PDB 6M8F as the search model. sMb5 pCaaF and sMb13 (*P*2_1_ and *P*2_1_2_1_2_1_) pCaaF data were collected at beamline 9-2 at the Stanford Synchrotron Radiation Lightsource, integrated and scaled with XDS^52^ and merged using AIMLESS.^53^ The sMb5(O2beY) and 6M8F structures were used for MR for the sMb5(pCaaF) and sMb13(pCaaF) (space group *P*2_1_) structures, respectively. The sMb5(pCaaF) structure was then used for determination of the sMb13(pCaaF) structure in space group *P*2_1_2_1_2_1_ by rigid body refinement. Restraint files for the unnatural amino acids were generated using PHENIX, and structures were refined using Coot^54^ and PHENIX.^55^ Anisotropic B-factor refinement was performed for the pCaaF structures. All structural figures were made using PyMOL Molecular Graphics System (Schrödinger, LLC). Polder omit maps were generated using PHENIX.^55^

### Rosetta Modeling of Stapled Myoglobin Variants with eUAAs

We first ran the Rosetta Match application^56^ to search for suitable locations where the staples can be placed. The whole staple moiety was treated as three separated residues, namely, a tyrosine-derived residue with a dangling bond at the para-position (three-letter code TYZ), a deprotonated cysteine with an unsatisfied valence on the sulfur atom (CYX), and a free staple ligand. For each staple, we defined the geometry of two bonds formed between the staple and TYZ, CYX in a Match constraint file.^57^ Then, the matcher application can search through the myoglobin protein backbone to fit the staple into positions where the stapling geometry can be satisfied. Two residues were mutated to TYZ and CYX, respectively, to accommodate the staple. For each type of eUAA-based staple, both the final stapled state and the S_N_2 transition state/Michael addition reaction intermediate were considered in the matcher searching step. The locations that were able to be identified in both states were deemed a hit. Then, we applied the Rosetta FastRelax protocol^58^ onto the matcher output to further refine their structures and obtained the energy scores. For pCaaF, pAaF, and pVsaF, we were able to identify both sMb2 and sMb5 constructs in the matching step, but the sMb7 hit was not found for all three staples because of the likely suboptimal fit of the stapling geometry with the backbone. Thus, we manually built a pCaaF-based sMb7 initial structure by superimposing the pCaaF staple onto the O2beY-based model of sMb7, followed by Rosetta Relax to obtain the final pCaaF-based sMb7 Rosetta model.

### Comparison of Energetically Feasible Conformations of Stapled, Unfolded Proteins

**Figure 2:**
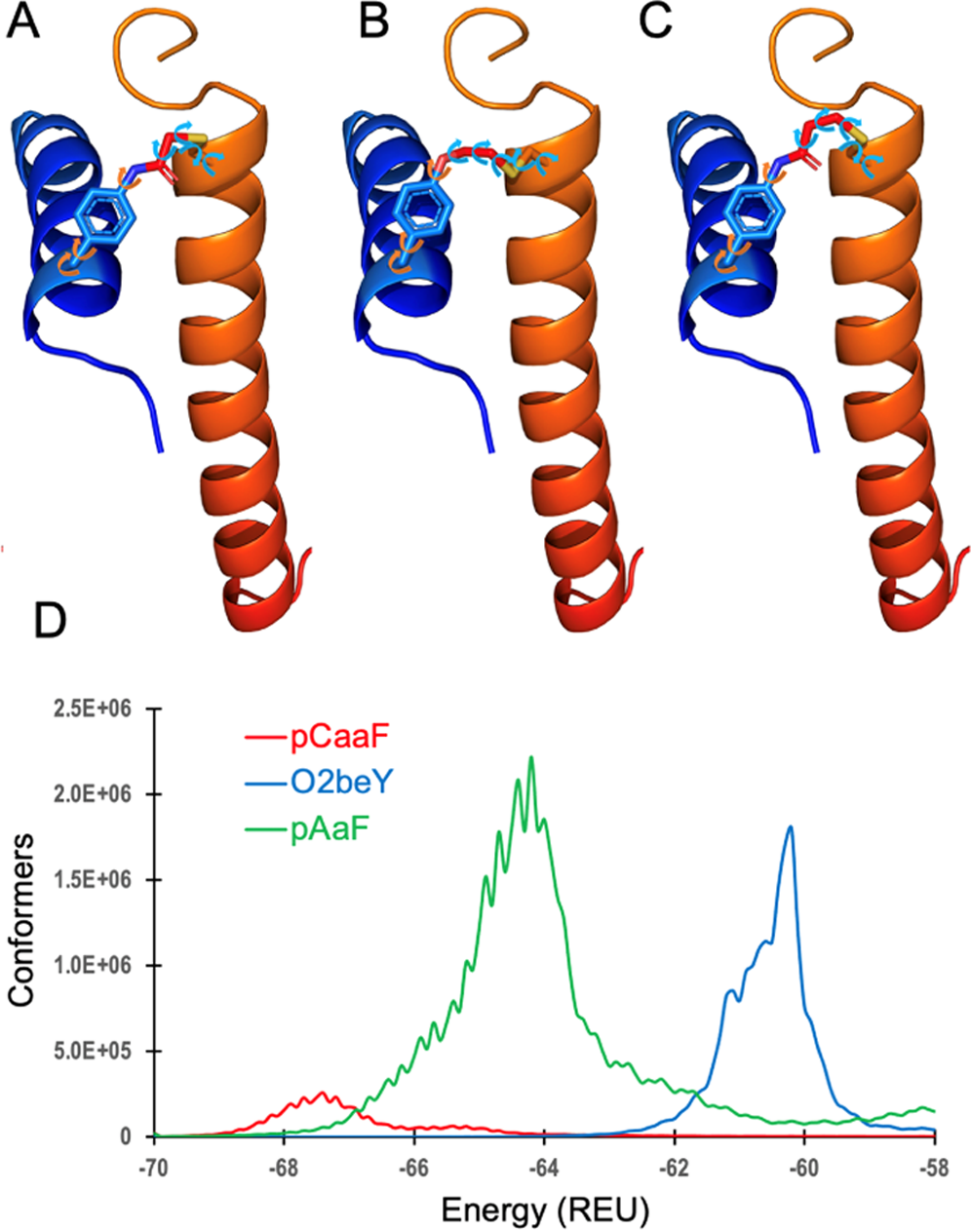
Models of (A) pCaaF-, (B) O2beY-, and (C) pAaF-based sMb5 used for the unfolded state density of state calculation. Sampled dihedral angles in each eUAA are indicated by arrows. (D) Unfolded state modeling of sMb5 with indicated eUAAs shows that the number of energetically feasible conformations as measured by Rosetta energy units (REUs) follows the order pCaaF < O2beY < pAaF. The pVsaF staple is not shown or included in the analysis.

To generate the conformational ensembles of the eUAA-stapled proteins in their unfolded state, we performed an exhaustive conformational search on the cross-linked pose (Figure 2A–C) by varying all rotational degrees of freedom within the staple but holding the rest of the protein backbone dihedrals fixed. All flexible bond dihedrals were sampled discretely with an interval of 30°, i.e., from (χ1, ..., χi, ..., χN) to (χ1, ..., χi ± 30, ..., χN). We explicitly sampled all possible combinations of the bond dihedral values and recorded the total energy as a function of those bond dihedral values. All generated conformations with an intramolecular repulsive score term (*fa_intra_rep*) higher than 22 Rosetta energy unit (REU) were discarded as unfeasible staple conformations. The obtained conformational ensemble size (binned by 0.2 REU) was plotted against the calculated energy to obtain the density of states corresponding to the different eUAA-based cross-links.

## Results and Discussion

### Selection of Protein Stapling eUAAs and Model Constructs

For this study, a sperm whale myoglobin variant Mb(H64V,V68A) with high activity and stereoselectivity for olefin cyclopropanation reactions via non-native carbene transferase activity^59−63^ was used as the model enzyme. The high stereoselectivity of this carbene transferase is dependent upon the configuration of the two mutated residues in the distal heme pocket, which enable the precise control of styrene attack to the heme–carbene reactive intermediate during catalysis.^64^ As such, this property can serve as a sensitive probe for detecting structural alterations within the enzyme active site that impact function.

Using Rosetta-guided design, we previously designed a set of nine Mb(H64V,V68A)-derived variants, called sMb1 through sMb9, in which a potentially stabilizing O2beY/Cys thioether staple (Figure 1B) was installed in various regions of the protein scaffold (Figure 1D–F).^47^ In these designs, the O2beY-based staple was designed to cross-link positions that are spatially proximal but separated in the primary sequence by 73–121 intervening residues (Figure 1D–F), while being accommodated in a strain-free configuration and/or promoting favorable interactions with the surrounding residues. Out of these nine designs, sMb2(O2beY) and sMb5(O2beY) were found to undergo efficient cross-linking, leading to an increase in the enzyme thermostability (Δ*T*_m_′ = +10.2 and +13.0 °C), as determined by thermal denaturation experiments using circular dichroism (CD) (Table 1, entries 2 and 6).^47^

In the interest of expanding the scope of this protein stapling approach to other types of eUAAs, we chose to investigate *para*-chloroacetamido phenylalanine (pCaaF), *para*-acrylamido phenylalanine (pAaF), and *para*-vinylsulfonamido phenylalanine (pVsaF) (Figure 1C) as alternative residues for covalent protein stapling. All of these eUAAs are genetically encodable, and they were recently found to mediate proximity-induced cysteine alkylation in the context of small cyclic peptides,^49^ although their functionality for intramolecular protein stapling was not investigated. pCaaF-mediated stapling was expected to lead to a thioether staple via cysteine-mediated nucleophilic substitution of the side-chain alpha chlorine group, whereas pAaF- and pVsaF-mediated stapling would occur via Michael addition of the cysteine-borne thiol group to the side-chain acrylamide and vinylsulfonamide moiety, respectively, in these eUAAs. The aforementioned sMb2 and sMb5 designs were chosen as initial test beds for comparing/contrasting the stapling efficiency of these eUAAs as well as their effects on thermostability compared to the previously characterized O2beY-based constructs. Computational modeling of these pCaaF-, pAaF-, and pVsaF-based constructs was carried out as a preliminary step to assess whether the staples could be accommodated without significant distortion of the native structure (Table S1; Figure S3).

### Expression and Characterization of sMb2 and sMb5 Constructs

All of the corresponding sMb2- and sMb5-derived variants could be produced as soluble proteins via recombinant expression in *E. coli* cells containing the appropriate orthogonal aminoacyl tRNA synthetase/tRNA_CUA_ pair for the genetic incorporation of pCaaF, pAaF, or pVsaF via amber stop codon suppression.^49,65^ The proteins were expressed in a tag-free form and purified via cation-exchange chromatography. Sodium dodecyl sulfate polyacrylamide gel electrophoresis (SDS-PAGE) analysis showed a higher electrophoretic mobility for all of the eUAA-containing proteins compared to the parent Mb(H64V,V68A) variant (Figure S4), which is consistent with a more compact unfolded state due to the presence of an intramolecular cross-link. This behavior is consistent with that of the previously characterized sMb2(OpgY) and sMb5(O2beY) constructs containing a O2beY/Cys staple.^47^ Interestingly, the pVsaF-containing constructs showed only partial stapling (∼50% based on gel densitometry), as indicated by the presence of a second band with electrophoretic mobility similar to that of the parent protein (Figure S4). On the other hand, sMb2(pCaaF) and sMb5(pAaF) constructs showed the presence of a minor species (∼20–30% based on gel densitometry) with a molecular weight of ∼30 kDa, which was assigned to a dimeric byproduct resulting from intermolecular cross-linking (Figure S4). UV–visible absorption spectroscopy showed that all of the constructs exhibit the characteristic Soret band (Figure S5), indicating the proper folding of these constructs in the heme-bound form.

**Figure 3:**
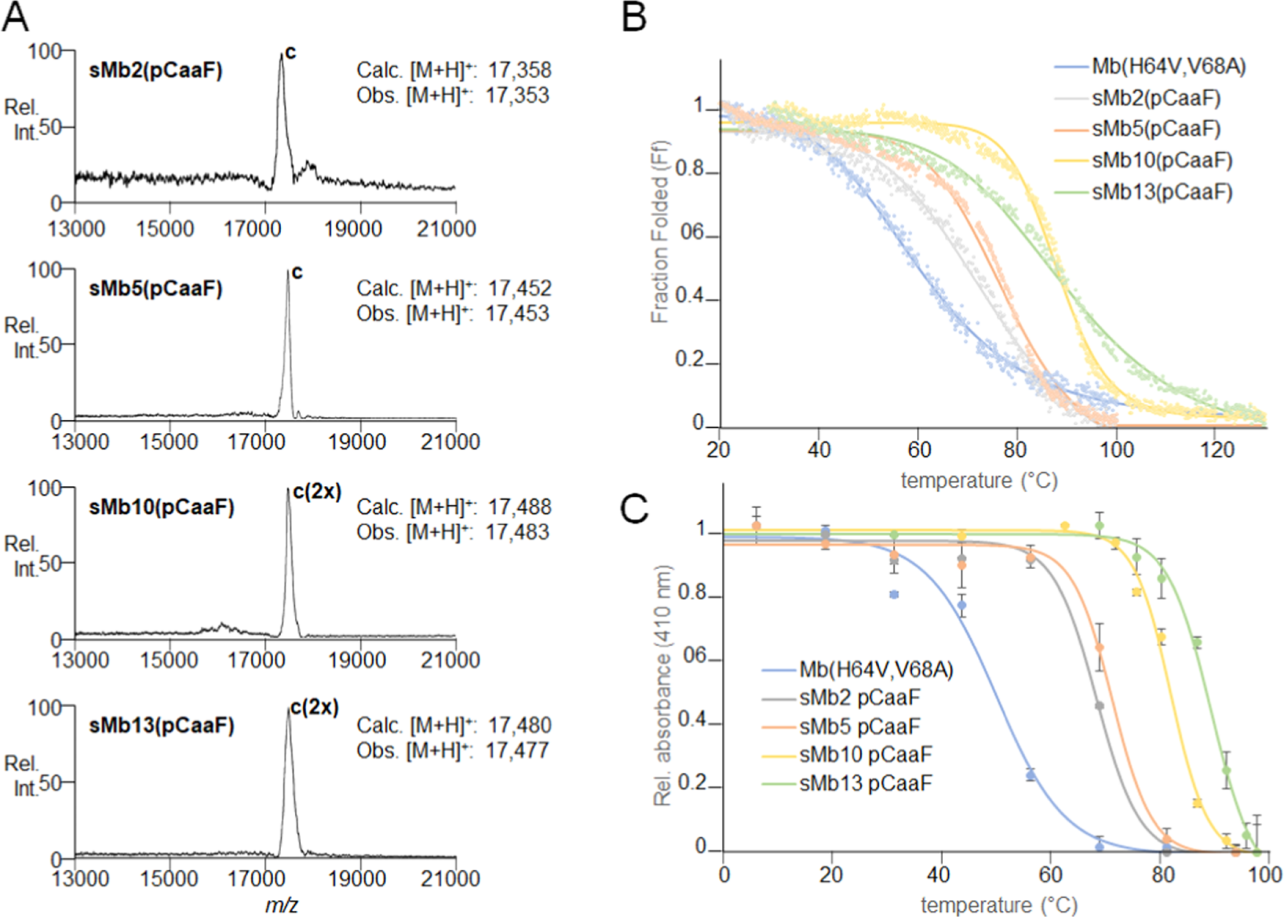
Characterization of pCaaF-stapled Mb variants (sMb(pCaaF) variants). (A) MALDI-TOF MS spectrum of representative sMb(pCaaF) variants: c, cross-linked; c(2×), doubly cross-linked. (B) Fitted thermal denaturation curves for Mb(H64V,V68A) and selected sMb(pCaaF) variants as measured via CD at 220 nm (*T*_m_′ determination). (C) Heat-induced inactivation curves (heme loss) for the same proteins as determined by the decrease of the Soret band signal (408 nm) after incubation (10 min) at variable temperatures (*T*_50_ determination). See Figures S1 and S2 and S7 and S8 for additional data. Rel. Int., relative intensity.

Further characterization of sMb2(pCaaF) and sMb5(pCaaF) using MALDI-TOF mass spectrometry (MS) showed single peaks corresponding to the expected masses of these proteins minus 36 Da, which is consistent with the loss of HCl (Figure 3A, top row). These results indicated the efficient and quantitative formation of the pCaaF/Cys staples in these constructs. MS analysis of the pAaF- and pVsaF-containing constructs also yielded masses that are consistent with the selective incorporation of pVsaF and pAaF into these proteins (Figure S6A). To probe the occurrence of the eUAA-mediated cross-linking reaction (which involves no change in molecular mass), these constructs were treated with iodoacetamide, with the formation of an alkylation adduct (+57 Da), revealing the presence of a free cysteine and thus the absence of the thioether staple. Consistent with the results from SDS-PAGE analysis, these experiments showed that the pAaF-based cross-links were formed efficiently and quantitatively, whereas the pVsaF-containing constructs underwent only partial stapling (Figure S6B). Altogether, these results demonstrated that all of the newly tested eUAAs, and, in particular, pCaaF and pAaF, are viable and efficient reagents for protein stapling in the system under investigation.

### Thermostability of Stapled Variants Using Different eUAAs

The thermostability of these Mb variants was examined by measuring their apparent melting temperatures (*T*_m_′) using circular dichroism (CD) (Figures 3B and S1). Consistent with previous results,^47^ the parent protein Mb(H64V,V68A) showed a melting temperature of 63.0 **°**C, while sMb2(O2beY) and sMb5(O2beY) were previously determined to have a higher *T*_m_′ by 10.2 and 13.0 °C, respectively (Table 1, entries 1, 2, and 6). Replacement of O2beY with pAaF in both the sMb2 and sMb5 designs led to more thermostable variants compared to that of the unstapled parent enzyme (Δ*T*_m_′ = +5.4–9.1 °C; Table 1, entries 3 and 7), albeit this stabilizing effect was inferior to that induced by the O2beY-based staple. A similar result was obtained for the sMb2(pVsaF) and sMb5(pVsaF) constructs, which showed 6–10 °C higher *T*_m_′ values compared to that for Mb(H64V,V68A) (Table 1, entries 4 and 8). Interestingly, sMb2(pCaaF) and sMb5(pCaaF) showed a dramatic increase in thermostability compared to the unstapled parent protein, corresponding to a Δ*T*_m_′ of +16.4 and +16.2 °C, respectively (Table 1, entries 5 and 9). Notably, for both constructs, the stabilizing effect of the pCaaF-based staple was significantly higher not only for the pAaF- and pVsaF-based staples but also with respect to the originally designed O2beY-containing constructs, as indicated by their 3.2–6.2 °C higher melting temperature (ΔΔ*T*_m_′) compared to the latter.

As a second measure of thermostability, half-maximal denaturation temperatures (*T*_50_) were determined by monitoring heme loss as reflected by the decrease in the Soret band (λ_max_: 409 nm) upon incubation of the hemoproteins at variable temperatures for 10 min (Figure 3C). This is a more stringent assay of thermostability since it monitors the ability of the sMb variants to remain associated with the heme cofactor, which is essential for their activity as carbene transfer biocatalysts. As thermal denaturation of myoglobin is irreversible under the applied experimental conditions, an increase in *T*_50_ signifies an increase in kinetic stability.^66^ In this assay, Mb(H64V,V68A) exhibits a *T*_50_ of about 61.1 °C (Table 1, entry 1), whereas sMb2(O2beY) and sMb5(O2beY) exhibit 2.7 and 10.4 °C higher *T*_50_ values, respectively (Table 1, entries 2 and 6). Intriguingly, the pVsaF-based staple conferred higher thermal stability in both constructs (sMb2(pVsaF): Δ*T*_50_ = +5.4 °C; sMb5(pVsaF): Δ*T*_50_ = +8.4 °C), whereas the pAaF-based staple increased the kinetic stability only for the sMb5 design (Δ*T*_50_ = +8.7 °C vs +1.6 °C for sMb2(pAaF)). In line with the results from the *T*_m_′ analyses, however, both sMb2(pCaaF) and sMb5(pCaaF) displayed the greatest increase in *T*_50_ values (Δ*T*_50_ = +13.5 and +15.8; Table 1, entries 5 and 9), providing additional evidence for the superior stabilizing effect induced by the pCaaF/Cys staple compared to that of the other eUAAs, including O2beY. Of note, in the context of sMb2, while the O2beY staple significantly increases the thermodynamic stability of the protein (*T*_m_′) but not its kinetic stability (*T*_50_), the pCaaF staple at the same position produces a dramatic and comparable increase in both *T*_m_′ and *T*_50_ (+16.4/+13.5 °C). When comparing the new eUAAs introduced here, it is also interesting to note that the stapled forms of sMb2(pAaF) and sMb5(pAaF) differ from their pCaaF-containing counterparts only by a methylene group at the level of the thioether staple. In spite of this small structural difference, sMb2(pCaaF) and sMb5(pCaaF) show significantly higher (i.e., +7–12 °C) thermostability in terms of both *T*_m_′ and *T*_50_. These results highlight the importance of subtle structural differences at the level of the staple that influence protein stability.

### Contribution of eUAA Conformational Entropy to Stabilization

Considering the structures of the eUAAs (Figure 1C) and the effect of stapling on the conformational entropy of the unfolded state,^47^ we hypothesized that for a given staple location (e.g., residues 5/126 in sMb5), stabilization should increase as the conformational flexibility of the cross-link itself decreases. For example, the amide bond in the pCaaF-Cys cross-link should result in considerable reduction in the conformational flexibility compared to that in O2beY, which has instead an ether moiety (leading to an additional rotatable bond). While the same amide group is also present in pAaF, the pAaF-Cys cross-link has one more rotatable bond compared to the pCaaF-Cys cross-link; this is expected to lead to increased conformational flexibility. Therefore, to measure the relative impact of the conformational flexibility of the cross-link itself on the unfolded state ensemble, we computationally examined the conformational properties of a model system composed solely of the helices A and H of myoglobin stapled at the sMb5 locations (residues 5/126) with each individual eUAA (Figure S3). The rest of the myoglobin chain is assumed to be sufficiently flexible and noninteracting in the unfolded state to permit all cross-linker geometries and therefore is not considered in this model. We explicitly enumerated all possible conformations of the cross-link (in 30 degree increments, the results are insensitive to the exact value of increment chosen in the simulation) while allowing the helices to orient freely and evaluated the energy of each configuration using Rosetta’s energy function (see Experimental Details). We find that the pCaaF ensemble has the lowest number of energetically feasible conformers, while pAaF and O2beY have a greater number of conformers (pCaaF < O2beY < pAaF; Figure 2) in the unfolded state model. Thus, stabilization is expected to follow the order sMb5(pCaaF) > sMb5(O2beY) > sMb5(pAaF) (Table 1), which agrees well with the experimentally observed values (Table 1).

### Effect of pCaaF-Mediated Stapling in other sMb Designs

Due to the superior stabilizing effect of the pCaaF staple in these initial constructs (i.e., sMb2 and sMb5), we extended these studies to a series of other sMb constructs, namely, sMb1, sMb3, sMb4, sMb7, and sMb9, which were previously designed to feature a solvent-exposed staple at varying positions across the protein scaffold^47^ (Figure 1D,E). Of note, these prior studies showed that, using O2beY as the cross-linking amino acid, efficient stapling was achieved only in sMb3, whereas it occurred partially in sMb4 and failed in the case of sMb1 and sMb9. These results were attributed to geometric constraints in the O2beY alkylation reaction by the cysteine residue, which required an optimal orientation of these residues for productive stapling.^47^ Thus, these designs provided a diverse set of test constructs in which protein stapling was accessible (sMb3), only partially accessible (sMb4), or inaccessible (sMb1, sMb9) using the previous O2beY-based methodology.

Upon expression in the presence of pCaaF, all of the target constructs were successfully isolated in soluble and properly folded (heme-bound) forms (Figure S7). Importantly, the characterization of the isolated constructs by MS and SDS/PAGE showed that they have undergone efficient stapling (Figures S8 and S9), including sMb1, sMb4, and sMb9, for which the formation of the O2beY/Cys staple was previously not possible. For sMb9, whose stapling with O2beY was unsuccessful, the use of pCaaF resulted in about 60% stapling efficiency as estimated by gel densitometry, whereas quantitative stapling was observed for all of the other constructs (Figure S9).

Thermal denaturation experiments further showed that pCaaF-mediated stapling was highly stabilizing in sMb1 (Δ*T*_m_ = +8.3 °C; Table 2, entry 2), sMb3 (Δ*T*_m_ = +11.4 °C; entry 4), and sMb9 (Δ*T*_m_′ = +7.2 °C; entry 8) and neutral in sMb4 and sMb7 (Δ*T*_m_′ = +1–2 °C; entries 5 and 7), with respect to the parent enzyme. Similarly to sMb2 and sMb5 (Table 2, entries 3 and 6), the pCaaF/Cys staple showed a superior stabilizing effect compared to the O2beY/Cys staple also in sMb3 and sMb4, whereas an opposite trend was observed for sMb7. A Rosetta model of sMb7(pCaaF) shows that this staple cannot be favorably accommodated without significant distortion of the structure (Figure S10; Table S1). Importantly, the higher stapling efficiency of pCaaF (over O2beY) also enabled us to establish that the computationally designed cross-links in 31/109 (sMb1) and 16/122 (sMb9) are thermostabilizing as originally predicted using our Rosetta-guided protein stapling approach.^47^ Also noteworthy is that for all of the constructs stabilized by the pCaaF staple, the increase in thermodynamic stability (*T*_m_′) was accompanied by an increase in kinetic stability, as reflected by the increase in *T*_50_ (Δ*T*_50_ = +6–16 °C). This trend also differs from the O2beY-containing counterparts, for which only two of the four constructs successfully stapled by this eUAA showed an increase in *T*_50_ in association with the increased *T*_m_′ (i.e., sMb2 and sMb5 but not sMb3 and sMb7; Table 2).

Overall, these results demonstrated that the O2beY and pCaaF staples could be readily interchanged in all constructs featuring a solvent-exposed staple, supporting the flexibility of these designs to accommodate alternative tyrosine-based stapling eUAAs. At the same time, they showed that pCaaF-mediated stapling is significantly more tolerant to the local environment of the staple compared to O2beY-mediated stapling. This result can be, in part, attributed to the higher electrophilic reactivity of the α-chloroacetamido moiety in pCaaF compared to that of the alkyl bromide group in O2beY.^49^ In addition to higher stapling efficiency, pCaaF-mediated cross-linking is able to induce a larger and more general (thermodynamic and kinetic) stabilizing effect on the hemoprotein compared to O2beY-based stapling. Thus, the pCaaF-based staple appears to provide a more efficient and robust tool for introducing cross-links within a protein scaffold.

### Doubly Stapled Mb Variants

Based on the results above, further thermostabilization of the Mb-based cyclopropanase was pursued by combining the covalent staples from the most promising variants, sMb2(pCaaF) and sMb5(pCaaF), to yield the doubly stapled variants sMb10(pCaaF) and sMb13(pCaaF). sMb10(pCaaF) combines the two cross-links between residues 36/109 (from sMb2) and 5/126 (from sMb5) in a single construct, whereas sMb13(pCaaF) features an additional “charge-compensating” mutation (H113E) to account for the removal of two solvent-exposed negatively charged residues (i.e., Glu109 and Asp 126) due to the introduction of these staples.^47^

After expression and purification from *E. coli*, MALDI-TOF MS analysis confirmed the quantitative formation of the two desired cross-links in both sMb10(pCaaF) and sMb13(pCaaF) (Figure 3A). Thermal denaturation analysis of these variants revealed that the combination of the two staples produces a large increase in both *T*_m_′ and *T*_50_ (Δ *T*_m_′: +26–27 °C; Δ*T*_50_: +24–30 °C; Table 2, entries 9 and 10) compared with the parent protein. These results indicate that the stabilizing effect of the two pCaaF/Cys cross-links is additive with respect to the singly stapled variants and that further stabilization in terms of kinetic stability (*T*_50_) is provided through the H113E mutation in sMb13(pCaaF) compared to that in sMb10(pCaaF). Importantly, these pCaaF-stapled Mb variants are also significantly more robust against thermal denaturation and temperature-induced heme loss compared to the O2beY-stapled counterparts, for which an ∼20 °C increase in *T*_m_′ and a 12–17 °C increase in *T*_50_ were previously measured.^47^ The melting temperature of these pCaaF-containing double-stapled variants approaches the boiling temperature of water (i.e., *T*_m_′ = 90 °C), a feature rarely achieved in previous enzyme engineering efforts and only after extensive efforts.^31,67^ The CD spectra of Mb(H64V,V68A), sMb2(pCaaF), sMb5(pCaaF), sMb10(pCaaF), and sMb13(pCaaF) were nearly superimposable, indicating that neither the pCaaF/Cys cross-link(s) nor the H113E mutation affected the protein secondary structure (Figure S11).

### Cyclopropanation Activity

**Figure 4:**
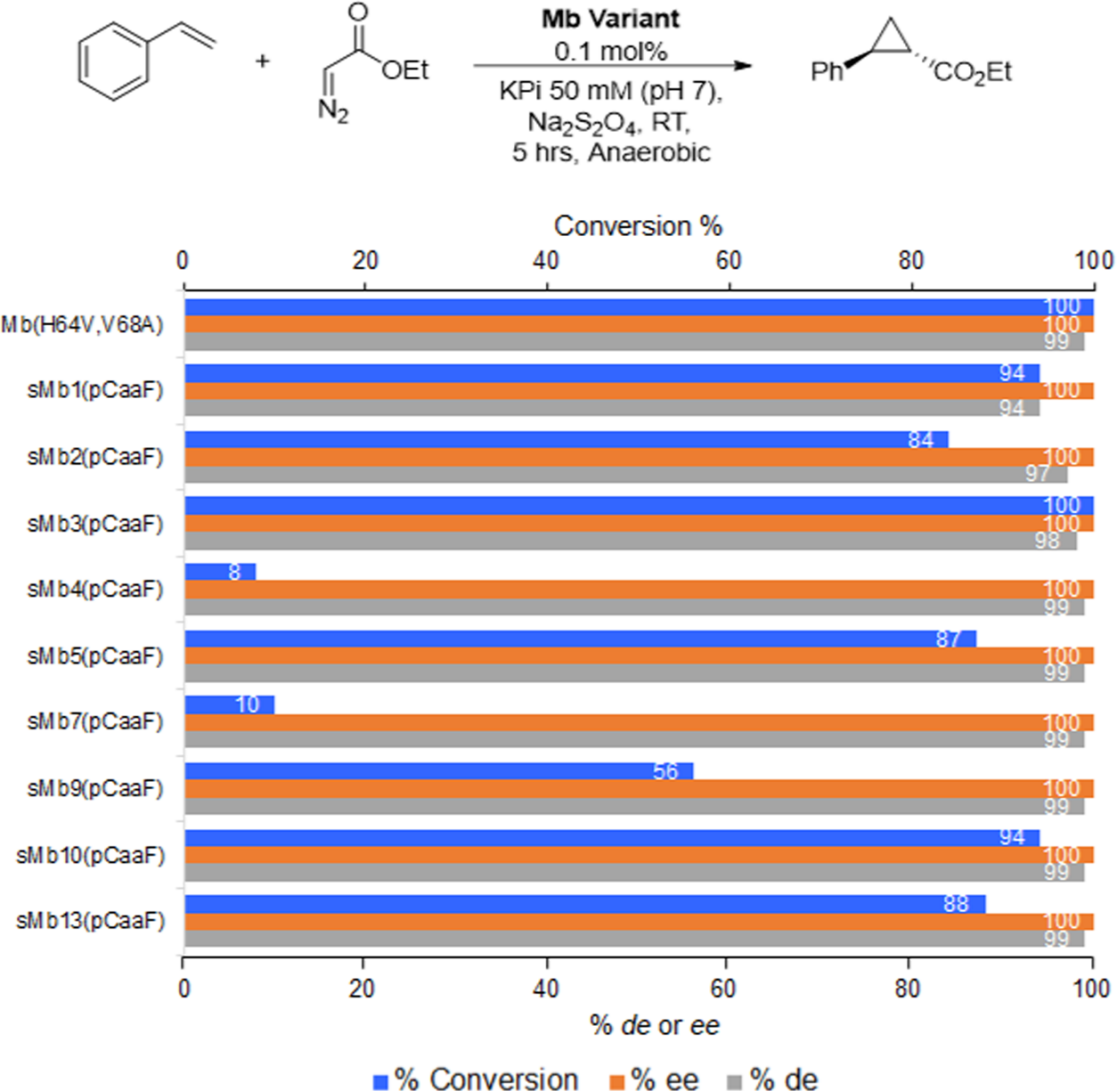
Activity and stereoselectivity of the pCaaF-stapled sMb variants in the cyclopropanation of styrene with EDA. Reaction conditions: 10 μM myoglobin, 10 mM styrene, 20 mM ethyl α-diazoacetate, and 10 mM sodium dithionite in KPi buffer (pH 7). Yields and diastereomeric/enantiomeric excess were determined by chiral GC using calibration curves generated with the authentic product.

To examine the impact of the pCaaF/Cys staple(s) on the catalytic activity and stereoselectivity properties of these proteins, the sMb(pCaaF) variants were tested in a cyclopropanation reaction with styrene and ethyl diazoacetate (EDA). Under the applied conditions (0.01 mol %), the parent enzyme catalyzes this reaction in quantitative yield with >99% *de* and *ee*. As shown in Figure 4, most of the singly stapled variants, including sMb2(pCaaF) and sMb5(pCaaF), and the two doubly stapled variants sMb10(pCaaF) and sMb13(pCaaF) showed high activity in this reaction, producing the cyclopropanation product in comparable or only slightly reduced yields (84–100%) compared to the parent enzyme. While remaining functional, sMb4(pCaaF) and sMb7(pCaaF) showed significantly reduced (10-fold) catalytic activity (8–10% yield) under identical conditions. Interestingly, the retention of parent-like catalytic activity was found to largely correlate with the staple being stabilizing in these constructs. Indeed, the only variants with reduced catalytic activity were sMb4(pCaaF) and sMb7(pCaaF), for which the staple has no stabilizing effect, and the partially stapled sMb9(pCaaF) variant (Figure 4). In all cases, including the most thermostabilized variants sMb2(pCaaF), sMb5(pCaaF), sMb10(pCaaF), and sMb13(pCaaF), the hemoproteins maintained a high level of diastereo- and enantioselectivities (94–99+% *de* and >99% *ee*), indicating that the staple(s) has no impact on the configuration of the active site, as required for mediating stereoinduction in this reaction.^64^

### Structural Characterization of Singly and Doubly Stapled Mb Variants

**Figure 5:**
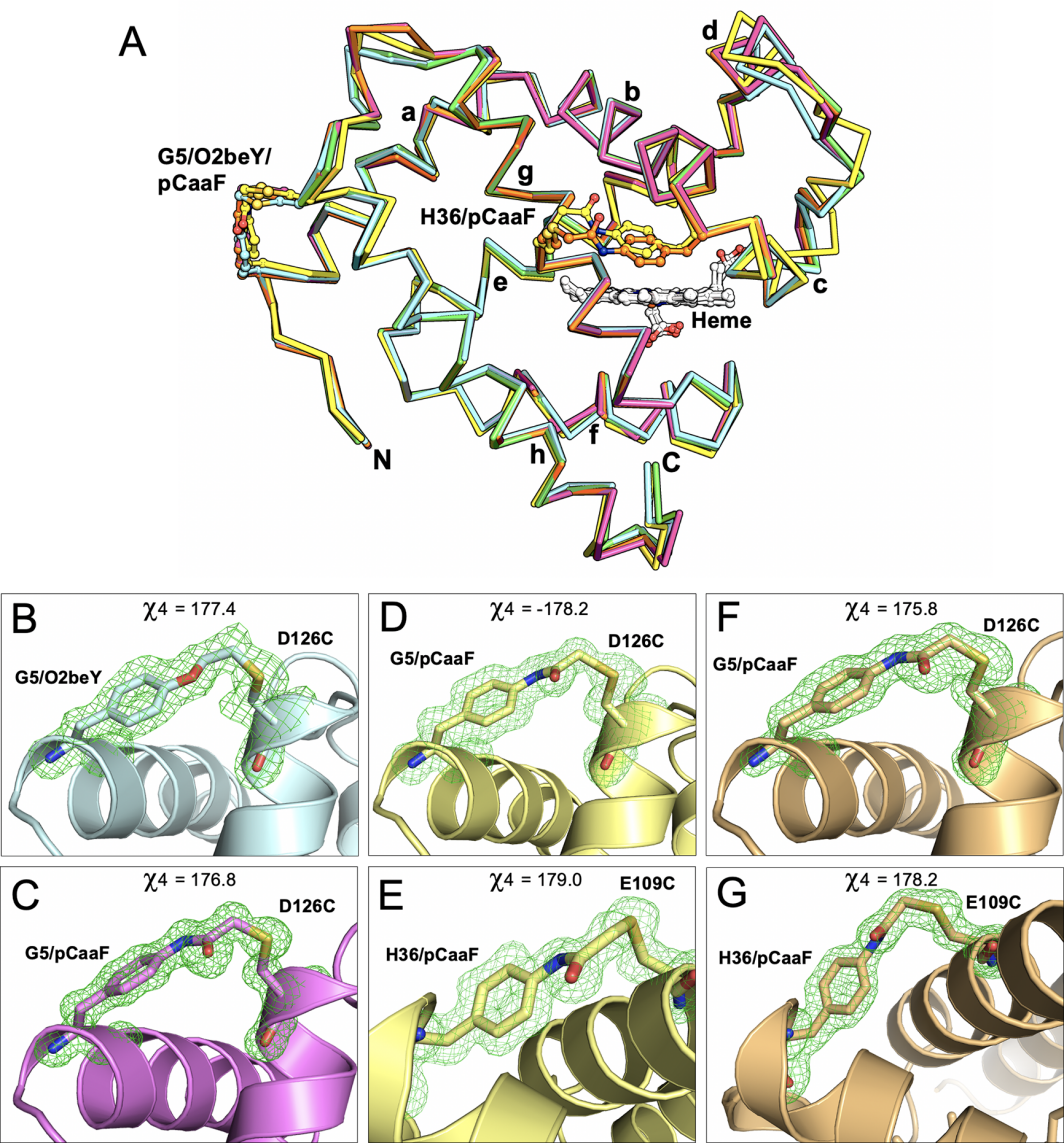
(A) Ribbon representation of superposed Mb(H64V,V68A) (green), sMb5(O2beY) (cyan), sMb5(pCaaF) (purple), sMb13(pCaaF) (*P*2_1_, yellow), and sMb13 (pCaaF) (*P*2_1_2_1_2_1_, orange) structures. Positions of staples and heme are shown in a ball-and-stick format. (B–G) Close-up views of staple in (B) sMb5(O2beY), (C) sMb5(pCaaF), (D) sMb13(pCaaF), staple 5/126 (*P*2_1_), (E) sMb13(pCaaF), staple 36/109 (*P*2_1_), (F) sMb13(pCaaF), staple 5/126 (*P*2_1_2_1_2_1_), and (G) sMb13(pCaaF), staple 36/109 (*P*2_1_2_1_2_1_). Nitrogen, oxygen, and sulfur atoms are shown in blue, red, and orange, while carbon atoms are the same color as the cartoon representation. Terminal dihedral angles (χ4) of O2beY and pCaaF residues are shown. Polder omit maps are shown in green mesh at 3σ.

To gain molecular insights into the effects of the engineered thioether staples on the structure and stability of the myoglobin protein architecture, we sought to determine crystallographic structures of the stapled variants, focusing on sMb5(pCaaF) and the doubly stapled sMb13(pCaaF) variant. For comparison between staples, the structure of sMb5(O2beY) was also pursued. We previously crystallized and determined the structure of the Mb(H64V,V68A) variant to a 1.1 Å resolution (PDB: 6M8F) in space group P6 using ammonium sulfate as the precipitant.^64^ Upon screening different crystallization conditions (see the Supporting Information (SI) for further details), sMb5(O2beY) and sMb5(pCaaF) were crystallized in space group *P*2_1_2_1_2_1_, whereas sMb13(pCaaF) was crystallized in both *P*2_1_2_1_2_1_ and *P*2_1_ space groups. Structures of sMb5(O2beY), sMb5(pCaaF), sMb13(pCaaF) (*P*2_1_2_1_2_1_ form), and sMb13(pCaaF) (*P*2_1_ form) were determined to resolutions of 1.7, 1.17, 1.3, and 1.3 Å, respectively (Figure 5A; Table S2). Structural alignment of the model backbone atoms yielded overall root-mean-square deviation (RMSD) values ranging from 0.17 to 0.47 Å (0.20 to 0.87 Å without outlier rejection), with the two pCaaF structures determined in the space group *P*2_1_2_1_2_1_ being the most similar to each other, while the sMb13 (*P*2_1_ form) structure is the most divergent (Table S3). Analysis of residue-by-residue RMSD plots reveals the majority of this deviation to be in the area around the CD loop and adjacent C and D helices, as well as the GH loop region (Figure S12).

Importantly, well-resolved electron density can be observed for the eUAAs and cross-linked cysteine residues, further demonstrating the formation of the thioether covalent bonds (Figure 5B–G). Refined bond lengths of 1.8 Å for all of the thioether bonds are observed except for the staple between residues 5 and 126 in sMb13(pCaaF) (*P*2_1_), which has a length of 2.1 Å. This difference may be due to strain on the staple imposed by interactions with the staple at residue 36 of a symmetry-related monomer (Figure S13A). However, terminal dihedral angles (χ4) are close to the ideal values of ±180° for all staples (Figure 5B–G), which is expected for the productive formation of the thioether staples given the projected trajectory of the nucleophilic attack in the bimolecular substitution reaction (S_N_2).^47^ Comparison of the crystal structures with our computationally designed models also demonstrated good agreement of the staple geometry when comparing the structures, underscoring the accuracy of the computationally predicted structures, although minor differences in the conformation of the staple moiety are observed for the *P*2_1_ structure, which may be due to the aforementioned crystal packing (Figure S14). The B-factor analysis also reveals that much of the differences between the crystal structures may be ascribed to crystal packing effects rather than the presence of the staples (Figure S15).

### Structural Factors Underlying Thermostabilization

**Figure 6:**
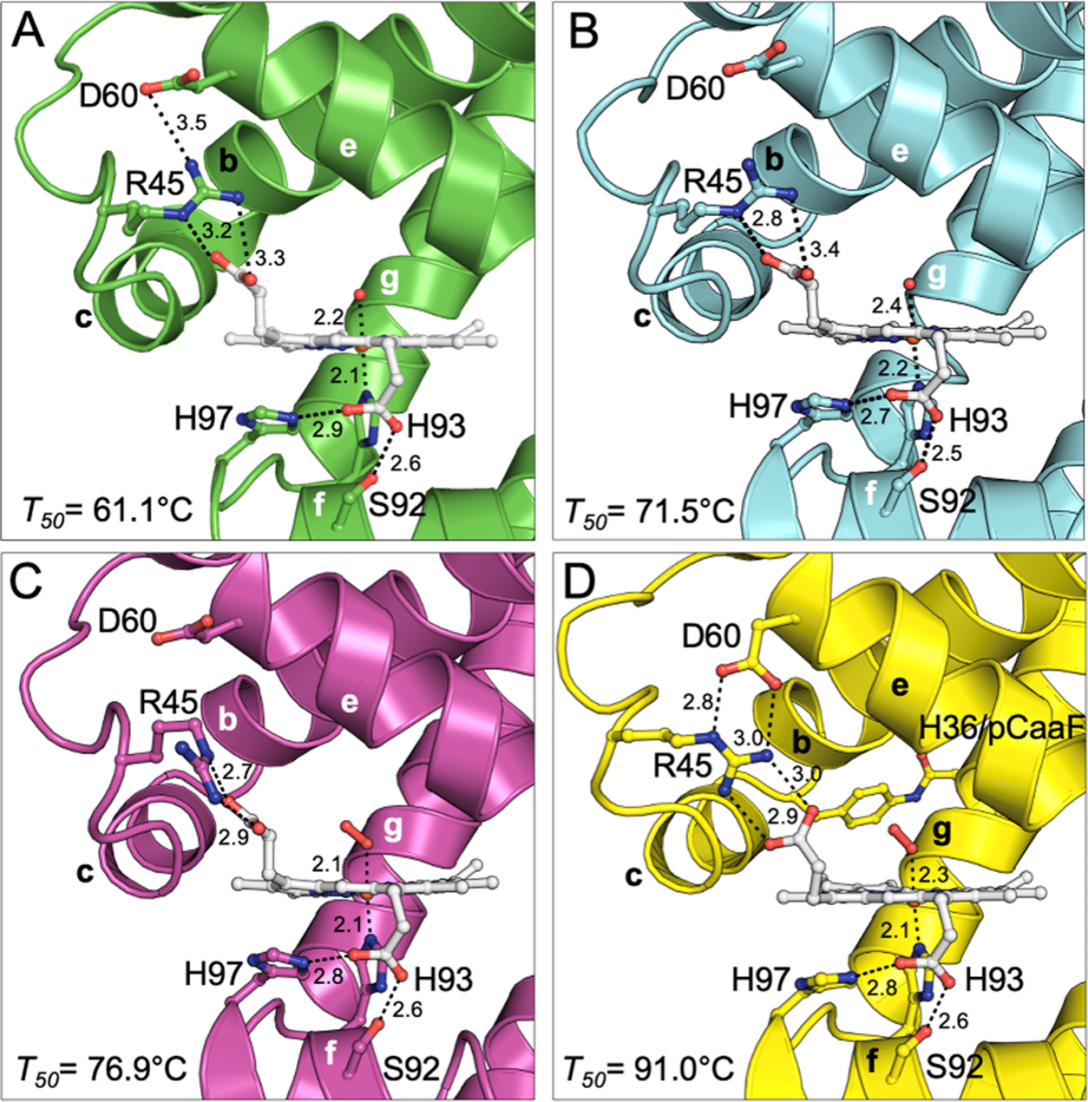
Myoglobin heme binding site and CD loop region. (A) Mb(H64V,V68A), (B) sMb5(O2beY), (C) sMb5(pCaaF), and (D) sMb13(pCaaF) (*P*2_1_). For the sMb13 structure in (D), the CD loop region containing Arg45 adopts a local geometry that differs from the other stapled structures, where Arg45 is stabilized through interactions with both the heme propionate group and Asp60. The pCaaF(H36)-C109 staple is also shown in panel (D). The sMb13(pCaaF) (*P*2_1_2_1_2_1_) model is not shown in this figure, but the structure adopts a very similar geometry to the sMb5(pCaaF) model (C). Experimental *T*_50_ values for each variant are also shown. Interatomic distances are shown in angstroms.

To further analyze the potential mechanisms behind stapling-induced thermostabilization in the Mb variants, we examined specific residues that may contribute to the stability of the heme-bound form of the protein, which is measured by the *T*_50_ values. As shown in Figure 6A–D, residue Arg45 was found to adopt variable positions in the structures, although pairwise interactions between Arg45 and the heme propionate group are seen in all of the structures. However, the Arg45 side chain appears most stable in the double-stapled sMb13(pCaaF) *P*2_1_ structure (Figure 6D) since it forms a well-ordered network of comparatively short hydrogen bonds or electrostatic interactions with both Asp60 and the heme propionate group. Although these bonds are also observed to an extent in the other structures, only the *P*2_1_ structure conserves a total of four bonds and B-factor analysis appears consistent with a higher stability for the region. For example, B-factors for the Arg45 side-chain nitrogen atoms involved in interactions with the oxygen atoms of the heme propionate group are (43, 47, 21, 21 Å^2^) for the parental structure and (18, 18, 20, 20 Å^2^) for the *P*2_1_ structure, respectively. The tight three-residue coordination could thus contribute, at least in part, to the greater thermostability of the doubly stapled variant compared to that of the single-staple variants, in particular, with respect to its ability to retain the heme at elevated temperatures, as reflected by its ∼15–20 °C higher *T*_50_ over sMb5(pCaaF) and sMb5(O2beY). The stabilizing network of heme–Arg45–Asp60 observed in the *P*2_1_ structure is not likely due to the direct participation of these residues in crystal packing since these residues do not come into close contact with symmetry-related chains (Table S4). However, it is possible that the structural perturbation due to the crystal contact made by the 36/109 staple (Figure S13A) is propagated through the C-helix and the CD loop region (featuring Arg45) (Figure S13B), leading to a conformational change of the CD loop, which, in turn, facilitates the formation of the stabilizing three-residue interaction network. Further computational and experimental characterization of sMb13 may reveal the extent to which this ternary interaction is populated in solution and how much it contributes to the observed enhanced stabilization of the protein.

Interestingly, in each of the three sMb(pCaaF) variants with the least improved stability and lower activity, sMb4, sMb7, and sMb9, a H-bond between two-residue side chains appearing in the Mb(H64V,V68A) crystal structure is likely disrupted by the staple residues or the additional substitutions in the vicinity of the installed staple (i.e., Arg118–Asp27 (2.8 Å) by sMb4 and sMb7 and His24–His119 (2.9 Å) by sMb9). The loss of a H-bond may offset part of the stabilization afforded by the staple. Indeed, sMb4, sMb7, and sMb9 are also among the several least stable O2beY-based sMb variants, since the salt bridge disruption is independent of the staple chemotype. Additionally, as noted above, stapling in sMb7 may induce backbone distortions (Figure S10; Table S1), thereby also contributing to its lower activity.

Another potentially important structural feature that leads to enhanced stability is a parallel π–π stacking interaction between the side chains of Phe106 and pCaaF36 that is observed in both sMb13 structures (Figure S13B). Previous experimental characterization of the sMb11 variant, which carries an F106A substitution compared to that of sMb10, suggested that this interaction contributes to the observed thermostabilization in constructs bearing the pCaaF36/C109 staple, which mimics the π–π stacking arrangement observed for the side chains of the highly conserved His36 and Phe106 residues in wild-type myoglobins.^47^ Together, our Rosetta analysis and crystallographic results suggest that the reduction in the unfolded state entropy and the stabilization of the folded state via new interactions both contribute to the increase in ΔG between the folded and unfolded states.

## Conclusions

In this study, we have demonstrated the functionality of an expanded set of electrophilic amino acids featuring chloroacetamido, acrylamido, and vinylsulfonamido side-chain groups for thermostabilization of an enzyme via computationally guided protein stapling. Covalent stapling through cysteine alkylation with *p*-chloroacetamido-phenylalanine (pCaaF) has proven particularly efficient and effective for this purpose, enabling efficient cross-linking at multiple sites across the protein scaffold and inducing larger thermostabilization effects in terms of both thermodynamic (*T*_m_′) and kinetic stabilities (*T*_50_). This strategy led to the development of a doubly stapled variant of a myoglobin-based cyclopropanase that features *T*_m_ and *T*_50_ values approaching the water boiling point (∼90 °C) and significantly enhanced resistance to thermal denaturation (Δ*T*_m_′ = +27 °C) and temperature-induced heme loss (Δ*T*_50_ = +30 °C) compared to the parent protein, with no deleterious impact on its catalytic function and stereoselectivity.

A key result of this work was the demonstration of the tunability of enzyme thermostability. Most remarkably, we showed that different staples can have a wide effect on thermostability even when they are introduced in the same location. In addition to enabling the computational design of stapled variants, Rosetta analysis revealed the importance of reduced conformational flexibility in pCaaF vs O2beY as a key factor contributing to the superior thermostabilizing effect of the former over the latter strategy. Furthermore, our crystallographic work provided additional insights into the structural determinants of thermostabilization of the folded state. These included rigidification of the protein core surrounding the cofactor along with promoting favorable noncovalent interactions between the staple and protein (e.g., π–π stacking interaction between Phe106 and pCaaF36) and between the protein and the cofactor (i.e., H-bonding and electrostatic interaction between Asp60 and Arg45 and heme propionate group). Our results further indicate that some regions of the myoglobin protein architecture remain variable in position and stability even in the presence of multiple thioether staples as in sMb13(pCaaF). While this is sufficient for achieving high levels of thermostabilization (+27–30 °C increase in Δ *T*_m_′ and Δ*T*_50_), these findings point at potential regions to be targeted via stapling or other strategies^32^ to achieve further thermostabilization in this scaffold as part of future studies.

Finally, this work provides valuable new insights into the use of thioether-based protein stapling as a robust strategy for achieving significant thermostabilization without sacrificing catalytic activity, an outcome that is rarely achieved in protein engineering. These findings lay the groundwork and provide valuable guidelines for the extension of this approach to other protein scaffolds, which will be the focus of future investigations.

## Supporting Information Available

Rosetta models (Figures S3 and S10; Table S1); spectroscopic, electrophoretic, and MS analysis of the stapled constructs (Figures S4–S9 and S11); CD thermal denaturation curves (Figures S1 and S2); analysis of crystallographic structures (Figures S12–S15; Tables S3 and S4), and crystallographic data and RMSD analysis (Table S2) (PDF)
Crystal structure PDB files (ZIP)

## Accession Codes

PDB: 7SPE, 7SPF, 7SPG, 7SPH.
