# Supplementary material for: Tuning Enzyme Thermostability via Computationally Guided Covalent Stapling and Structural Basis of Enhanced Stabilization
Source: Biochemistry. 2022 May 25;61(11):1041–54. doi: 10.1021/acs.biochem.2c00033 (PMC9178789; doi:10.1021/acs.biochem.2c00033)
Supplement: Supplementary file 1 — bi2c00033_si_001.pdf [file bi2c00033_si_001.pdf]

Supporting information for

Tuning Enzyme Thermostability via Computationally Guided Covalent Stapling and Structural Basis of Enhanced Stabilization

Jacob A. Iannuzzelli[a,‡], John-Paul Bacik[b,‡], Eric J. Moore[a], Zhuofan Shen[c], Ellen M. Irving[a], David A. Vargas, Sagar D. Khare[c,*], Nozomi Ando[b,*], Rudi Fasan[a,*]

[a] Department of Chemistry, University of Rochester, Rochester, NY 14627, United States

[b] Department of Chemistry and Chemical Biology, Cornell University, Ithaca, NY 14853, United States

[c] Department of Chemistry and Chemical Biology, Rutgers University, Piscataway, NJ 08854, United States.

‡ Equal contribution.

Table of Contents:

Supplementary Figures S1-S15 Pages S2-S20

Crystallization Experimental Procedure Page S21

Supplementary Tables S1-S4 Pages S22-S25

Figure S1. Thermal denaturation curves for sMb2 and sMb5 variants containing pCaaF, pAaF, or pVsaF. For each variant, a single set of raw data (θ_{MRE}) is shown along with extrapolated signals for folded (θ_f) and unfolded (θ_u) protein and the fitting curve (θ_{fit}).

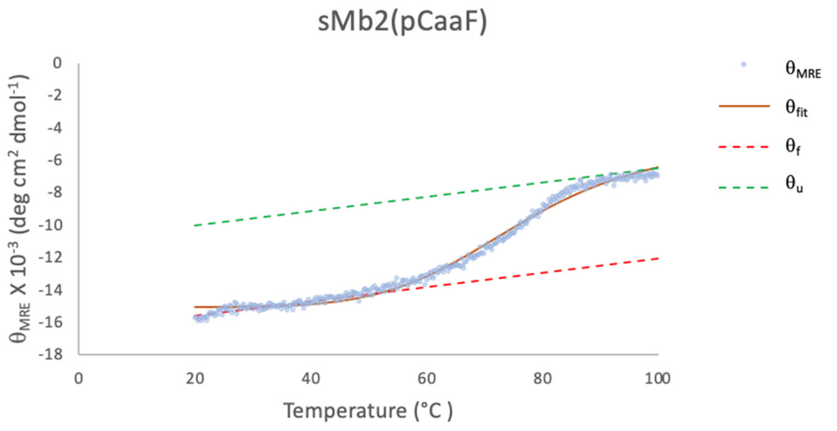

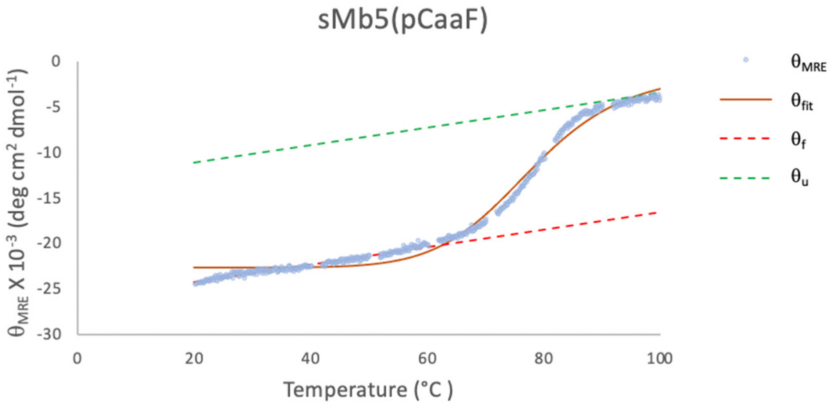

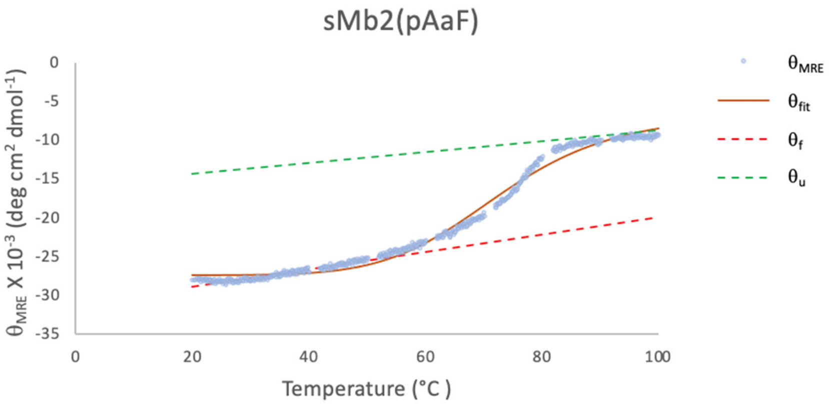

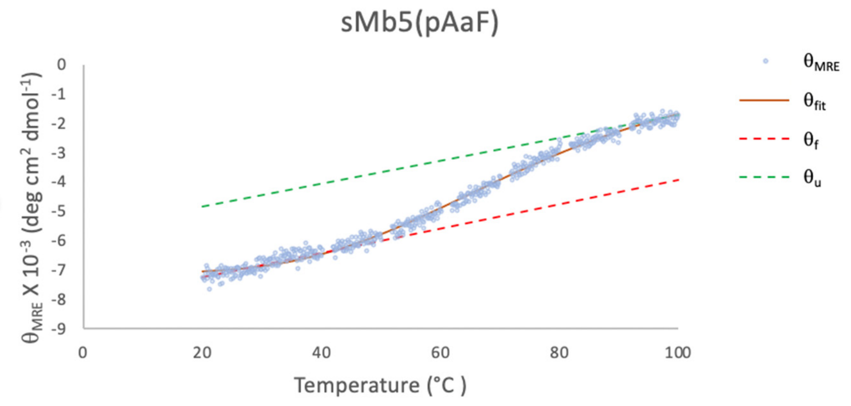
sMb5(pAaF)
θMRE
θfit
θf
θu
θMRE X 10-3 (deg cm2 dmol-1)
Temperature (°C)

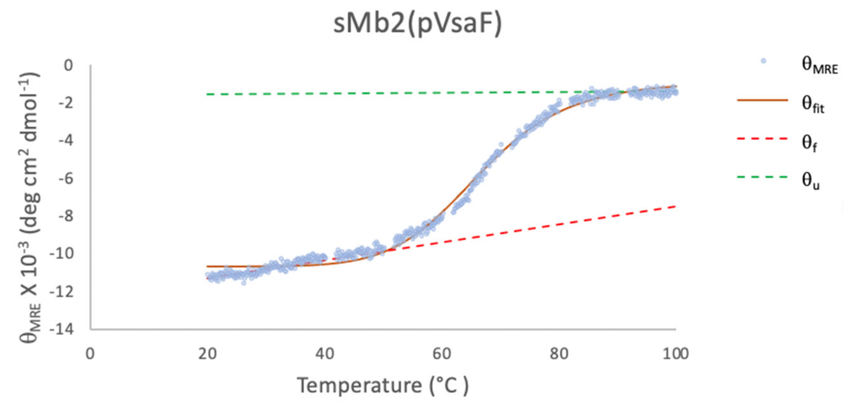
sMb2(pVsaF)
θMRE
θfit
θf
θu
θMRE X 10-3 (deg cm2 dmol-1)
Temperature (°C)

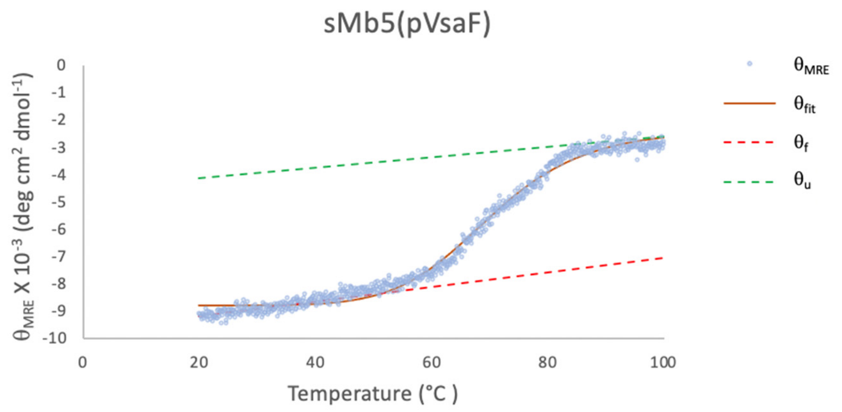
sMb5(pVsaF)
θMRE
θfit
θf
θu
θMRE X 10-3 (deg cm2 dmol-1)
Temperature (°C)

Figure S2. Thermal denaturation curves for pCaaF sMb variants. For each variant, a single set of raw data (θ_{MRE}) is shown along with extrapolated signals for folded (θ_f) and unfolded (θ_u) protein and the fitting curve (θ_{fit}).

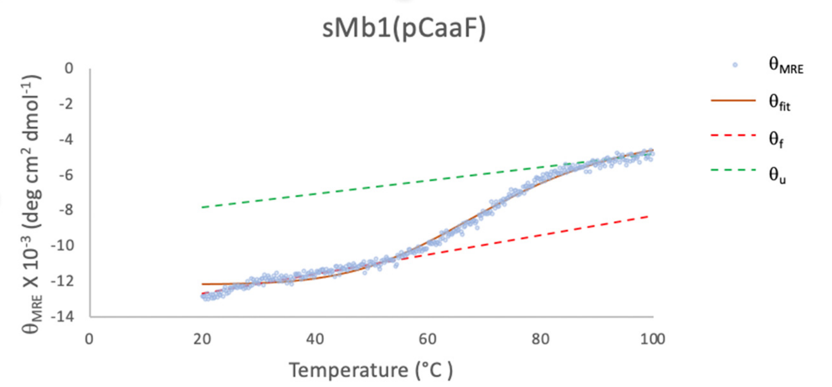

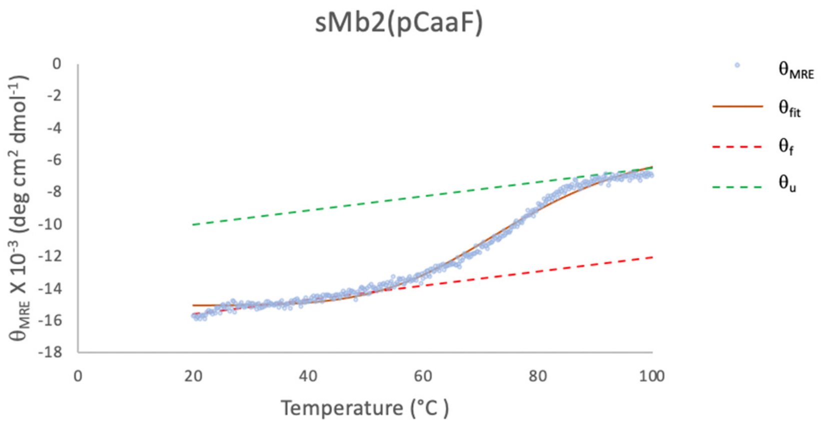

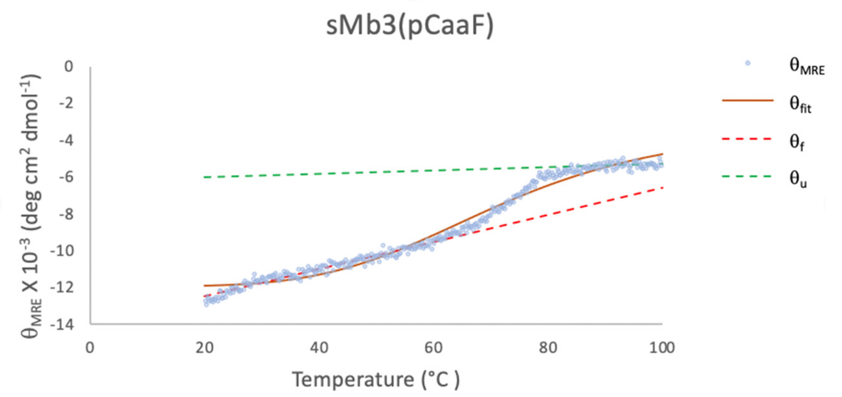

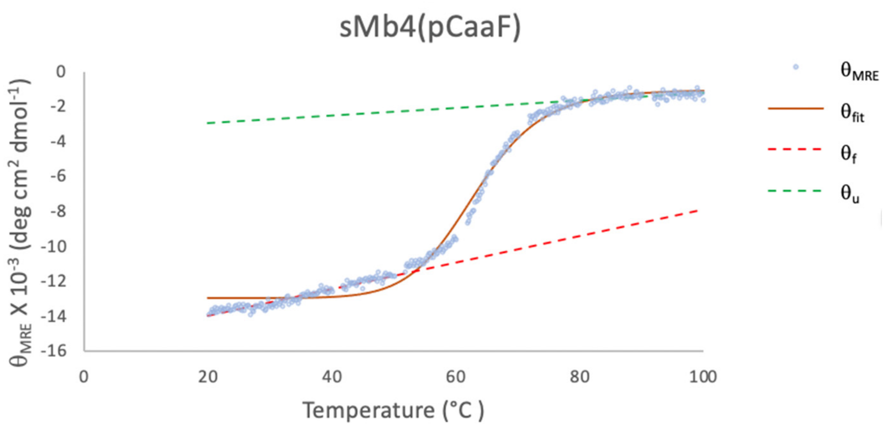
sMb4(pCaaF)
θMRE X 10-3 (deg cm2 dmol-1)
Temperature (°C)
θMRE
θfit
θf
θu

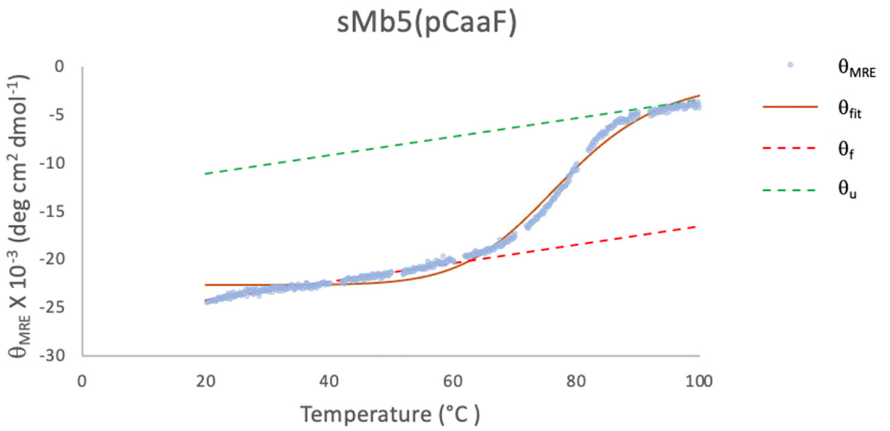
sMb5(pCaaF)
θMRE X 10-3 (deg cm2 dmol-1)
Temperature (°C)
θMRE
θfit
θf
θu

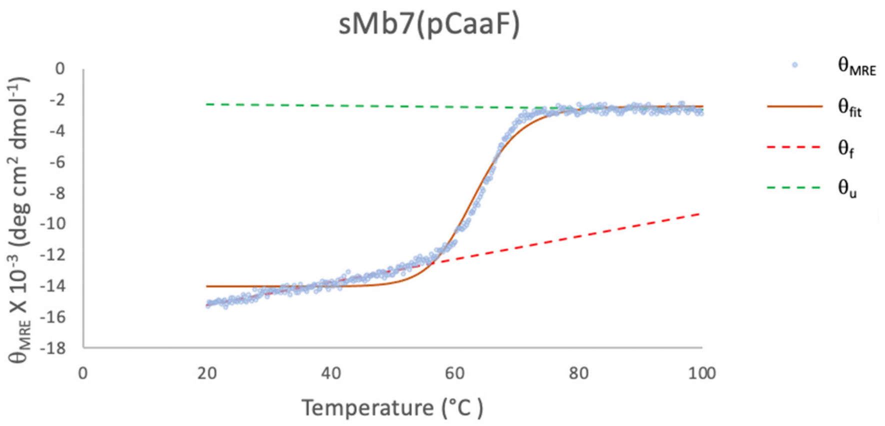
sMb7(pCaaF)
θMRE X 10-3 (deg cm2 dmol-1)
Temperature (°C)
θMRE
θfit
θf
θu

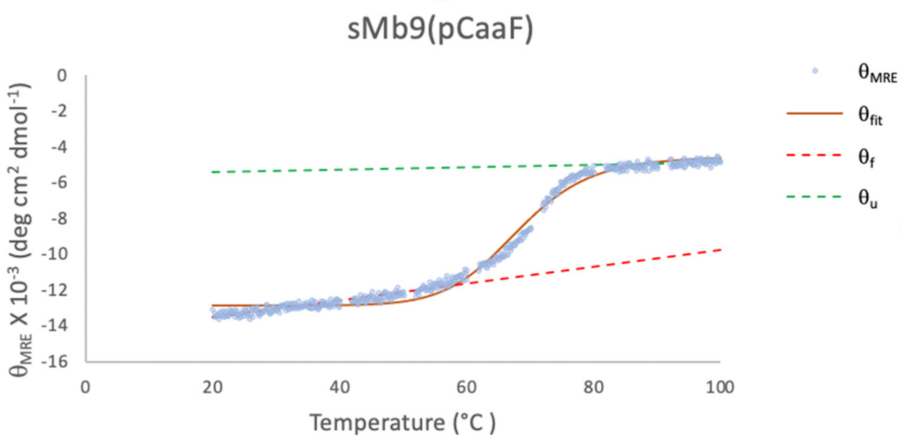

sMb9(pCaaF)
θMRE
θfit
θf
θu
θMRE X 10-3 (deg cm2 dmol-1)
Temperature (°C)

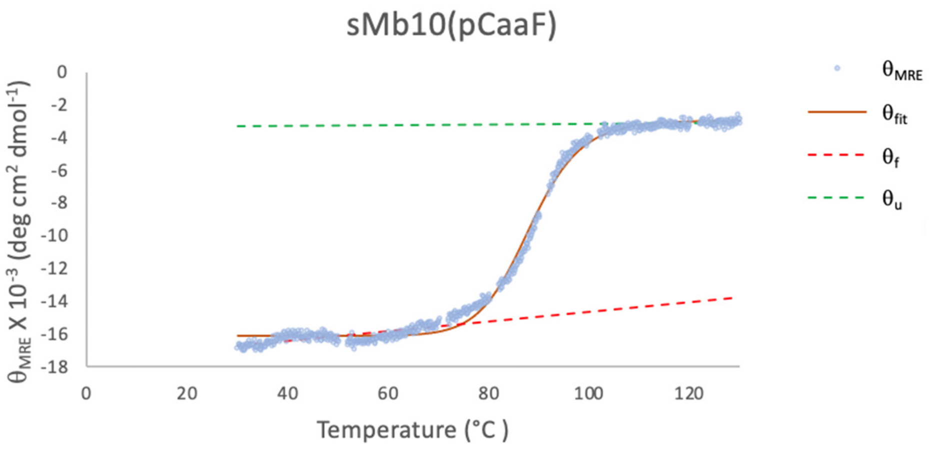

sMb10(pCaaF)
θMRE
θfit
θf
θu
θMRE X 10-3 (deg cm2 dmol-1)
Temperature (°C)

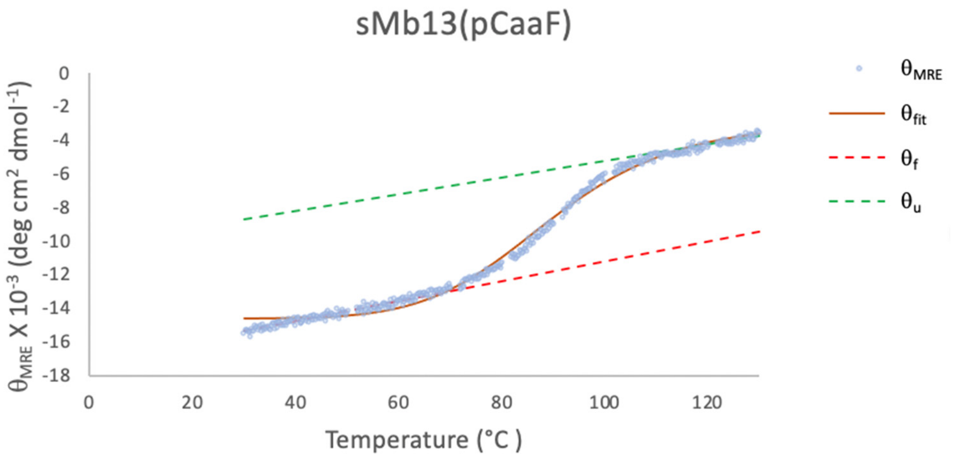

sMb13(pCaaF)
θMRE
θfit
θf
θu
θMRE X 10-3 (deg cm2 dmol-1)
Temperature (°C)

Figure S3. Rosetta models of stapled myoglobin variants sMb2 and sMb5. (**A**) pCaaF-, (**B**) pAaF- and (**C**) pVsaF-based sMb2, (**D**) pCaaF-, (**E**) pAaF- and (**F**) pVsaF-based sMb5. See also **Table S1** for energies.

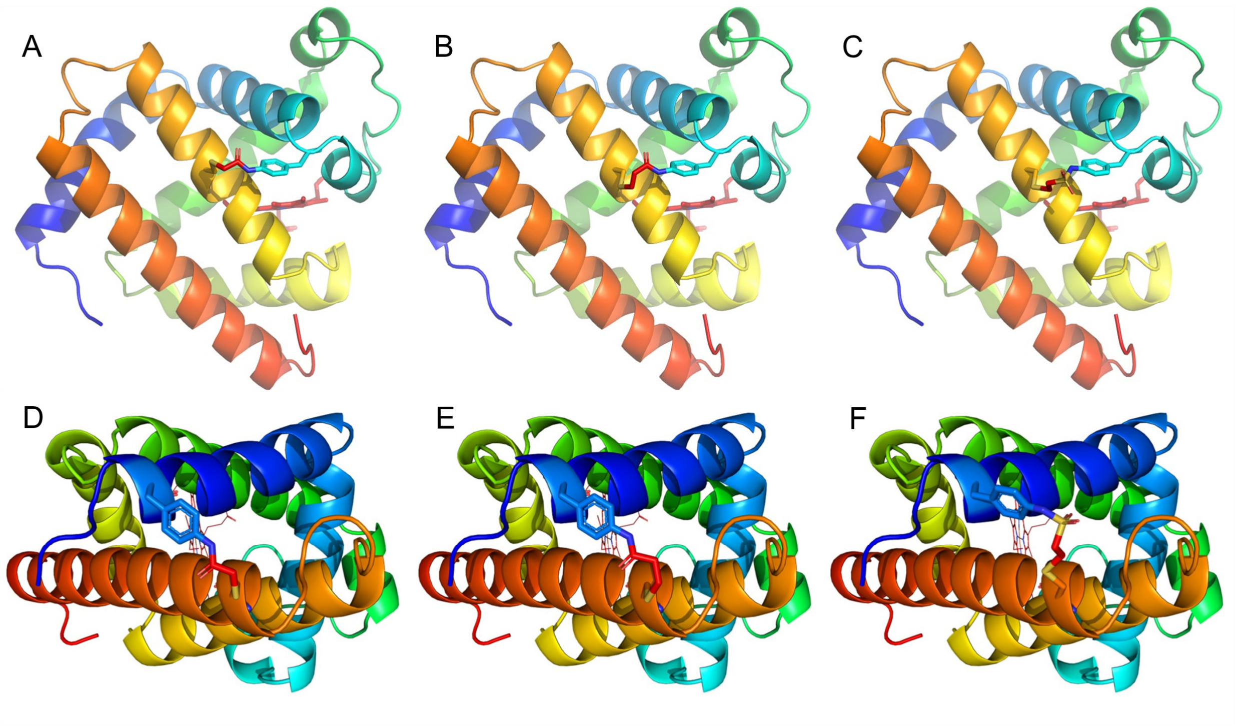

Figure S4. SDS-PAGE gel analysis (reducing conditions) of sMb2 and sMb5 variants containing pCaaF, pAaF, and pVsaF. The gel shows increased electrophoretic mobility for all constructs compared to the parent protein Mb(H64V,V68A), which is indicative of the presence of the eUAA/Cys crosslink as determined by mass spectrometry. The faint band at 35 kDa in the Mb(H64V,V68A) sample corresponds to a minor impurity from the purification steps. The pVsaF-containing constructs also show a band with parent-like electrophoretic mobility, indicating partial stapling (~50% based on gel band densitometry). sMb2(pCaaF) and sMb5(pAaF) show a small amount of dimer (22% and 29%, respectively, based on gel band densitometry) likely resulting from intermolecular eUAA/Cys crosslinking. MW of the proteins is ~17 kDa.

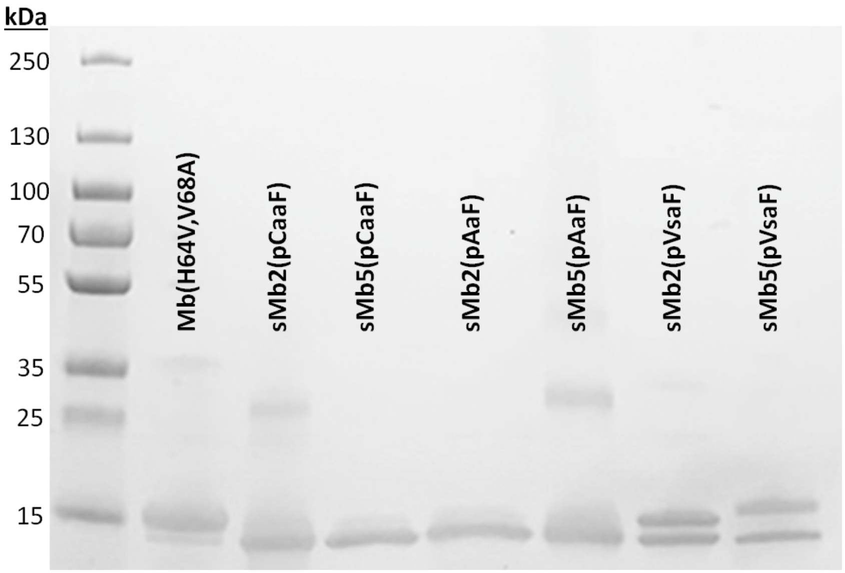

Figure S5. Visible-range electronic absorption spectra for the sMb2 and sMb5 variants containing (A) pCaaF, (B) pAaF, and (C) pVsaF, in the ferric (~410 nm) and ferrous forms (~434 nm).

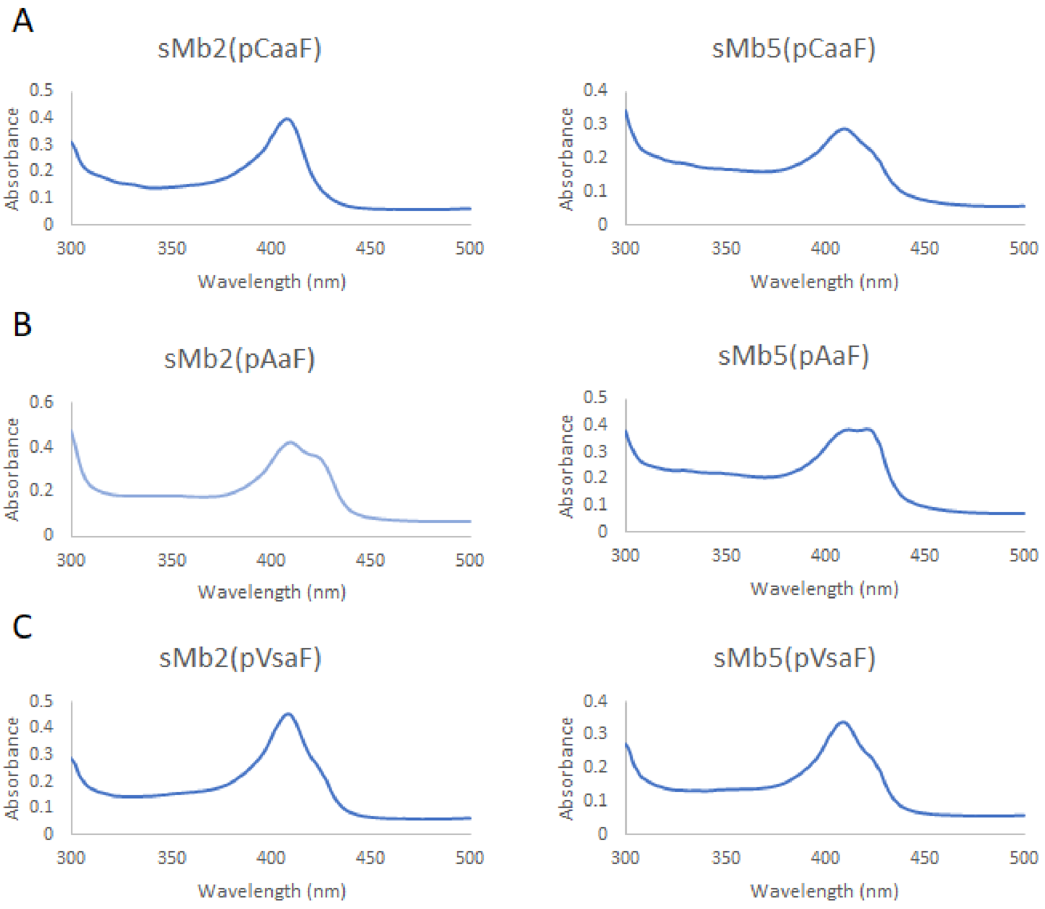

Figure S6. (A) Raw (*left*) and deconvoluted (*right*) MALDI-TOF MS spectra of the pAaF- and pVsaF-containing variants**.** Observed and calculated masses corresponding to the proton adduct ($[M+H]^+$) of the protein species are indicated. ‘c’ = crosslinked; ‘nc’ = not crosslinked. (B) Iodoacetamide alkylation experiment with sMb2(pVsaF) indicating partial stapling. No iodoacetamide alkylation was observed for the pAaF-containing constructs.

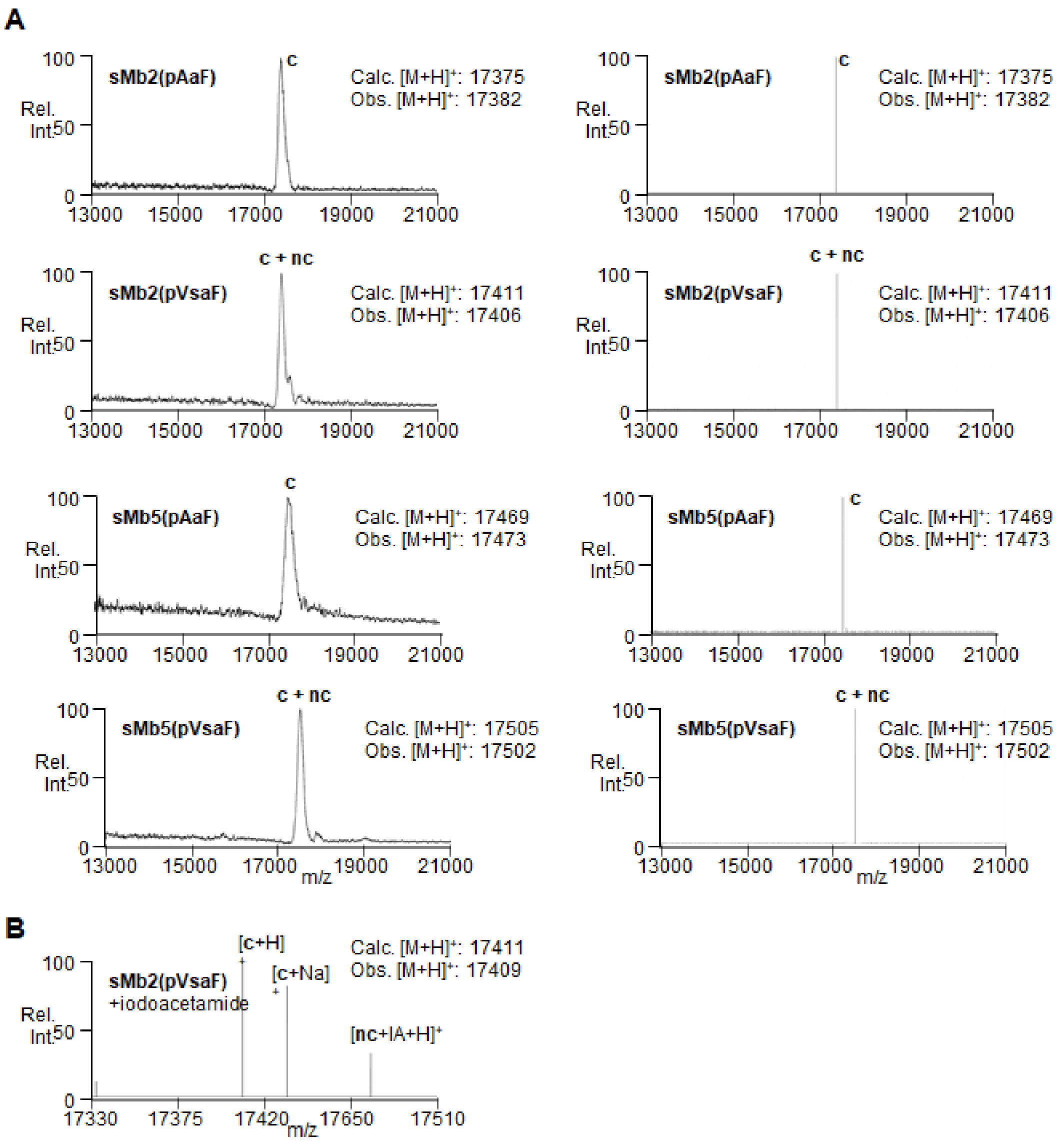

Figure S7. Visible-range electronic absorption spectra for pCaaF-containing sMb variants in the ferric (~410 nm) and ferrous forms (~434 nm).

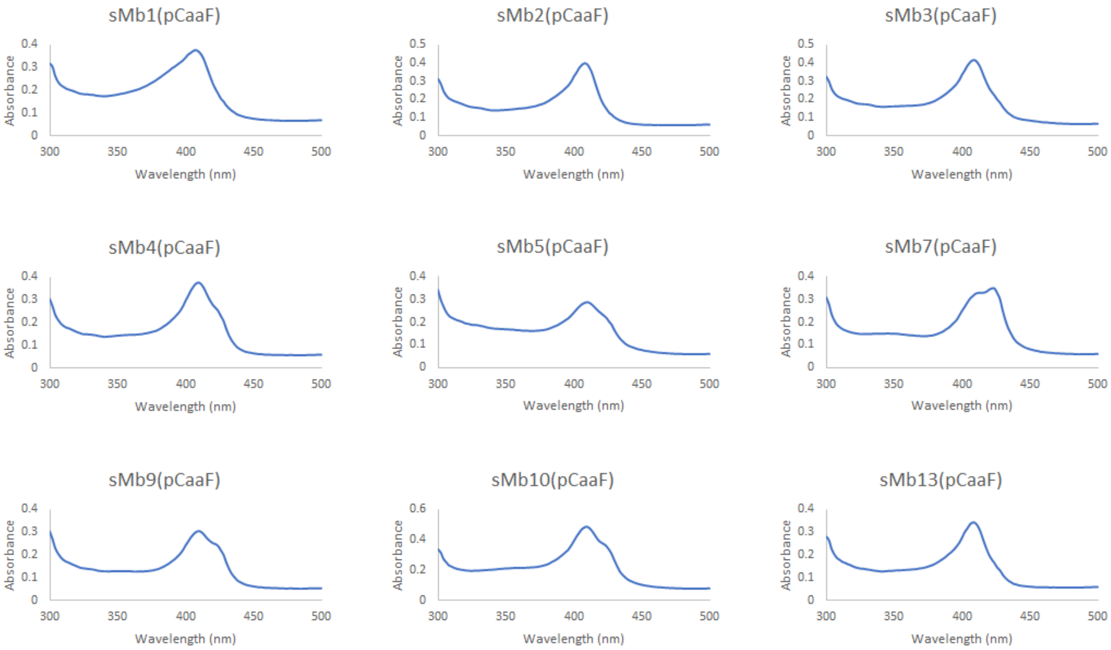

Figure S8. Raw (*left*) and deconvoluted (*right*) MALDI-TOF MS spectra of pCaaF-containing sMb variants. Observed and calculated masses corresponding to the proton adduct ($[M+H]^+$) of the protein species are indicated ('c' = crosslinked; c(2x) = doubly crosslinked). Peaks corresponding to the unstapled form of the protein were not observed.

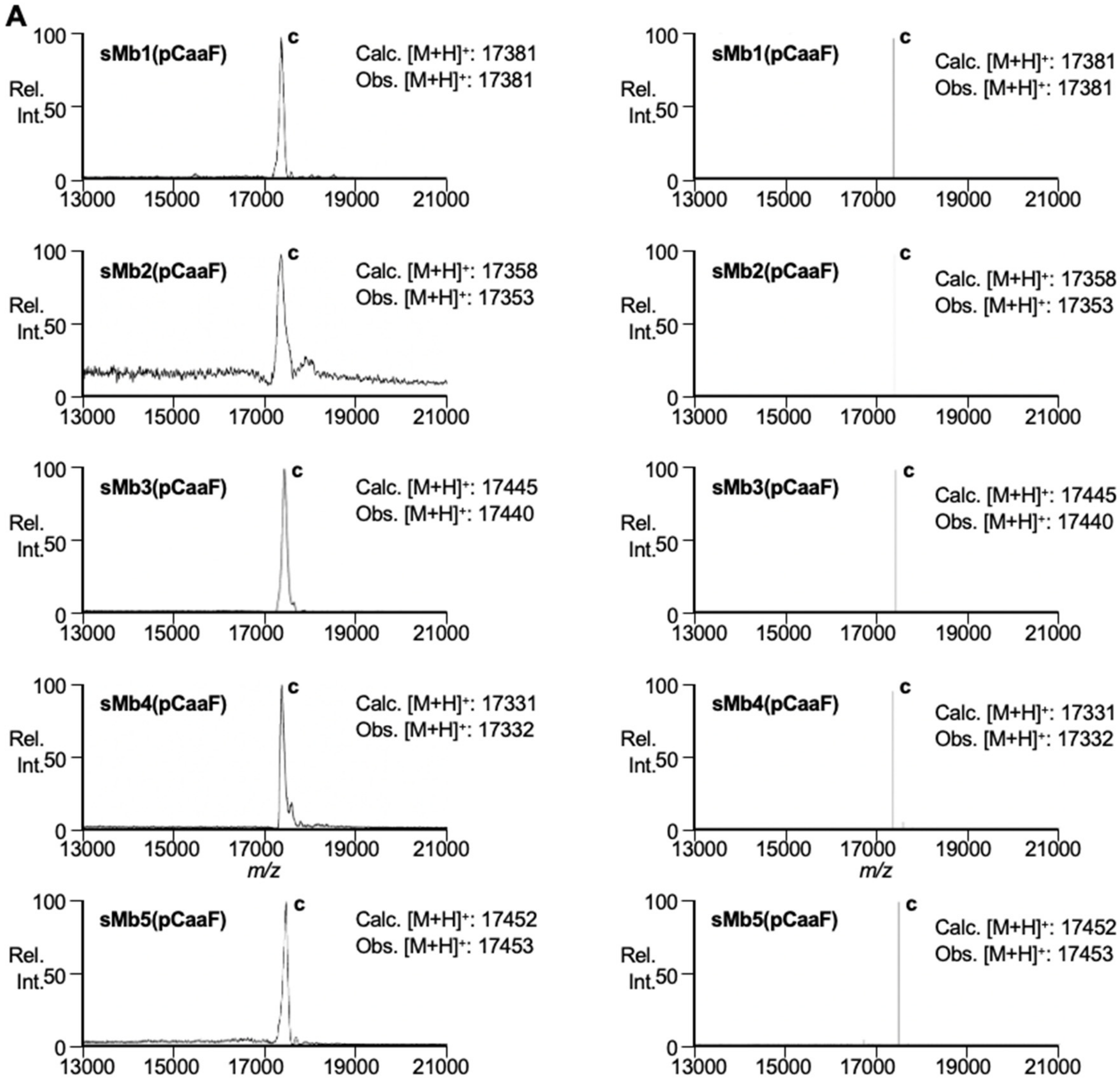

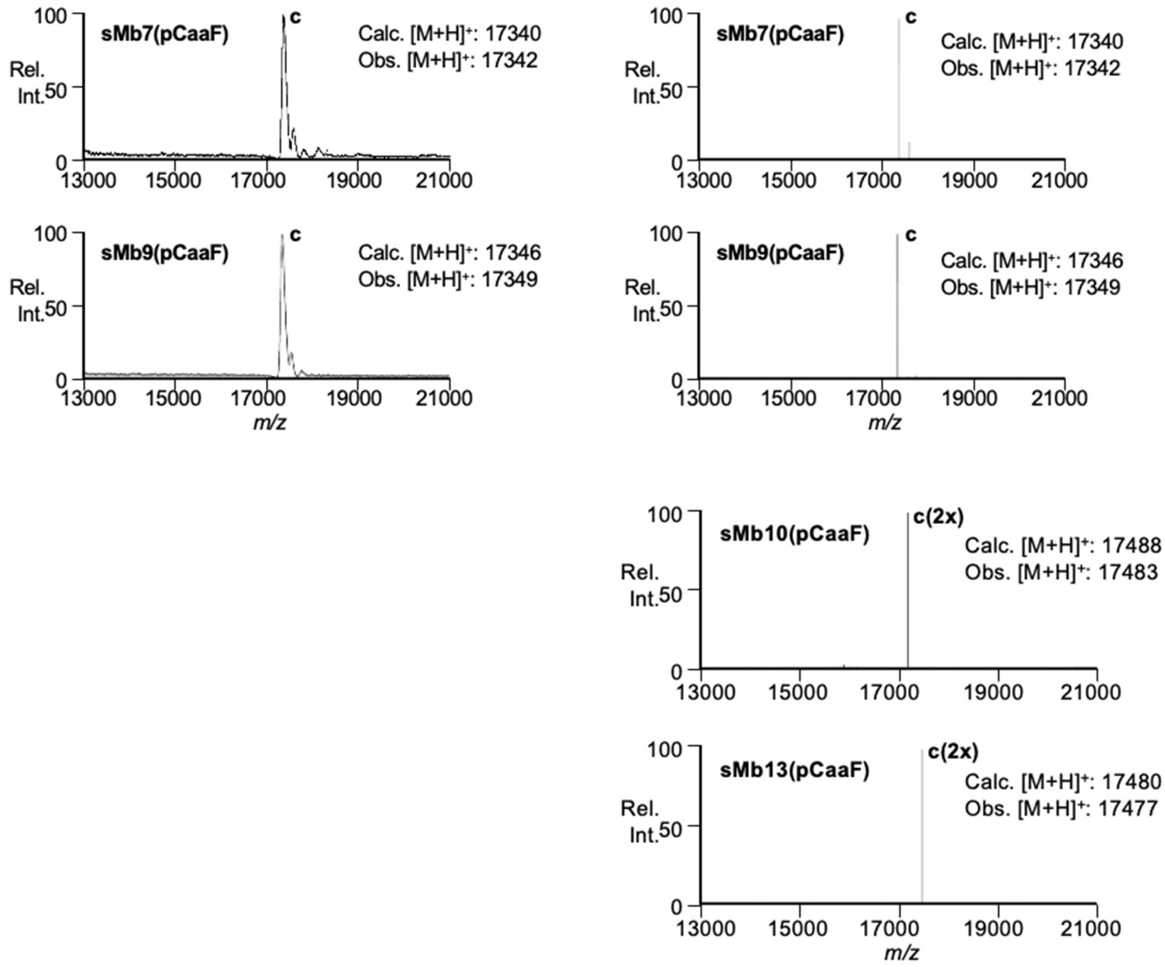
sMb7(pCaaF)
c
Calc. [M+H]+: 17340
Obs. [M+H]+: 17342
sMb9(pCaaF)
c
Calc. [M+H]+: 17346
Obs. [M+H]+: 17349
sMb7(pCaaF)
c
Calc. [M+H]+: 17340
Obs. [M+H]+: 17342
sMb9(pCaaF)
c
Calc. [M+H]+: 17346
Obs. [M+H]+: 17349
sMb10(pCaaF)
c(2x)
Calc. [M+H]+: 17488
Obs. [M+H]+: 17483
sMb13(pCaaF)
c(2x)
Calc. [M+H]+: 17480
Obs. [M+H]+: 17477
Rel. Int.
m/z

Figure S9. SDS-PAGE gel analysis (reducing conditions) of all pCaaF-containing sMb constructs except for sMb2(pCaaF) and sMb5(pCaaF), which are shown in **Figure S4**. The gel shows quantitative stapling for all constructs with the exception of sMb9, which shows partial crosslinking (~60% based on gel densitometry). The faint band at 35 kDa in the Mb(H64V,V68A) sample corresponds to a minor impurity from the purification steps.

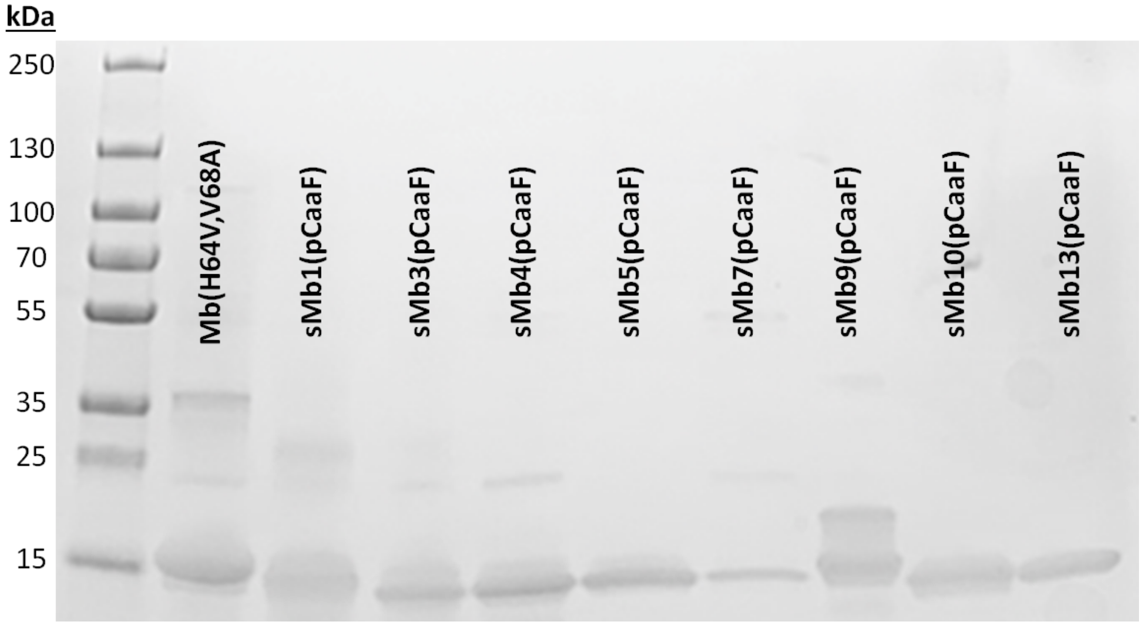

Figure S10. Rosetta models of stapled myoglobin variants sMb7(O2beY) (**A**) and sMb7(pCaaF) **(B)**. The stapling geometry is sub-optimal in sMb7(pCaaF) as visible in the distorted C—N bond(*). This lack of ideal geometry is also reflected in the constraint scores (**Table S1**).

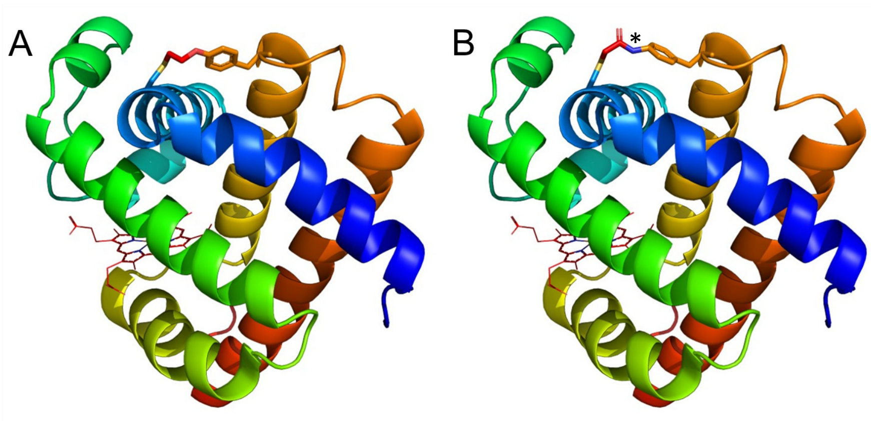

Figure S11. Overlay of near-UV circular dichroism spectra corresponding to Mb(H64V,V68A) and stapled variants sMb2(pCaaF), sMb5(pCaaF), sMb10(pCaaF), and sMb13(pCaaF).

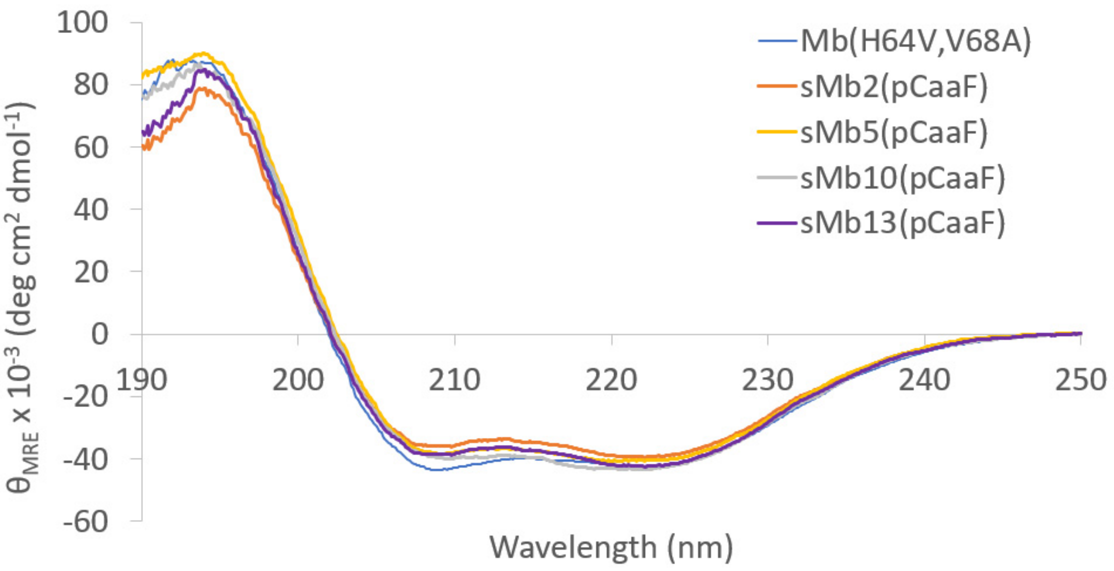

Figure S12. Residue by residue plots of RMSD values for each structure compared to every other structure in this study. Structure main chain atoms (n+ca+c+o) were first aligned and then the script RmsdByResidue (pymolwiki.org/index.php/RmsdByResidue) was used to determine RMSD of all main chain atoms of each residue pair. Horizontal axes are amino acid residue number and vertical axes are RMSD values.

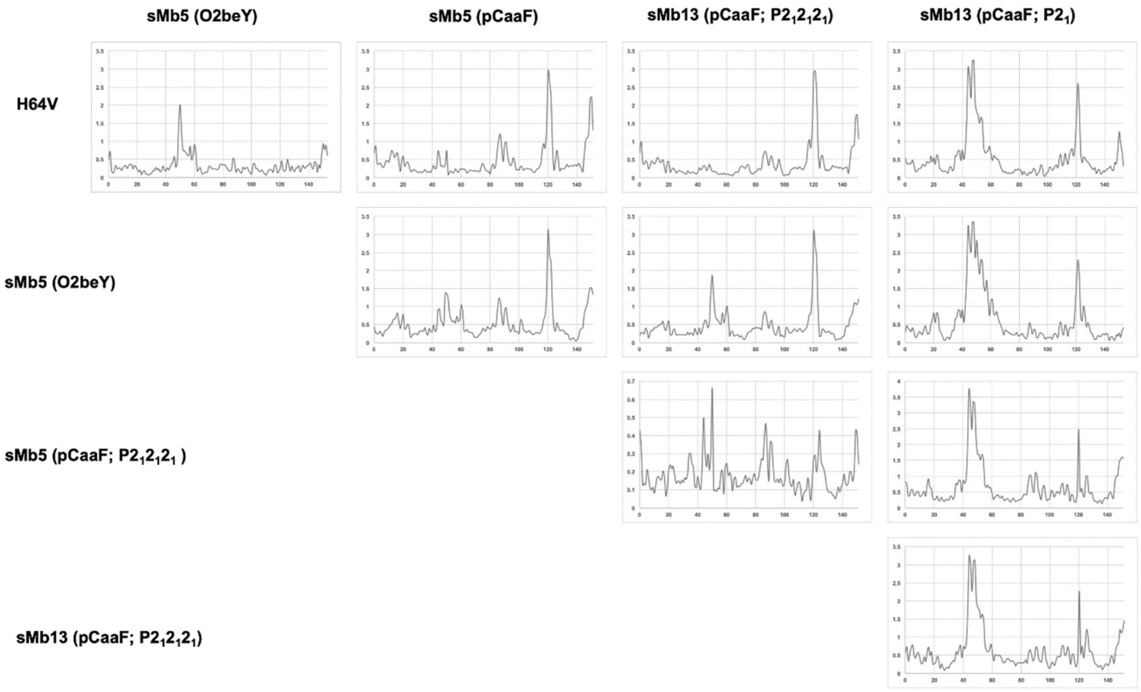

Figure S13. A) The two staples in sMb13 ($P2_1$) stack against each other in symmetry related monomers in the crystal. The symmetry related S3 and the mutated E113 also form a hydrogen bond. **B)** The pCaaF staple that forms a thioether bond between residue 36 and 109 adopts slightly different conformations in the two sMb13 structures. The positions of F106, heme and the CD loop are also shown. The distance between the center of the planes between the pCaaF ring and F106 is 4.3 Å for the $P2_1$ structure and 3.8 Å for the $P2_12_12_1$ structure. The sMb13 $P2_1$ structure is shown in yellow and the $P2_12_12_1$ structure is in orange cartoon representation.

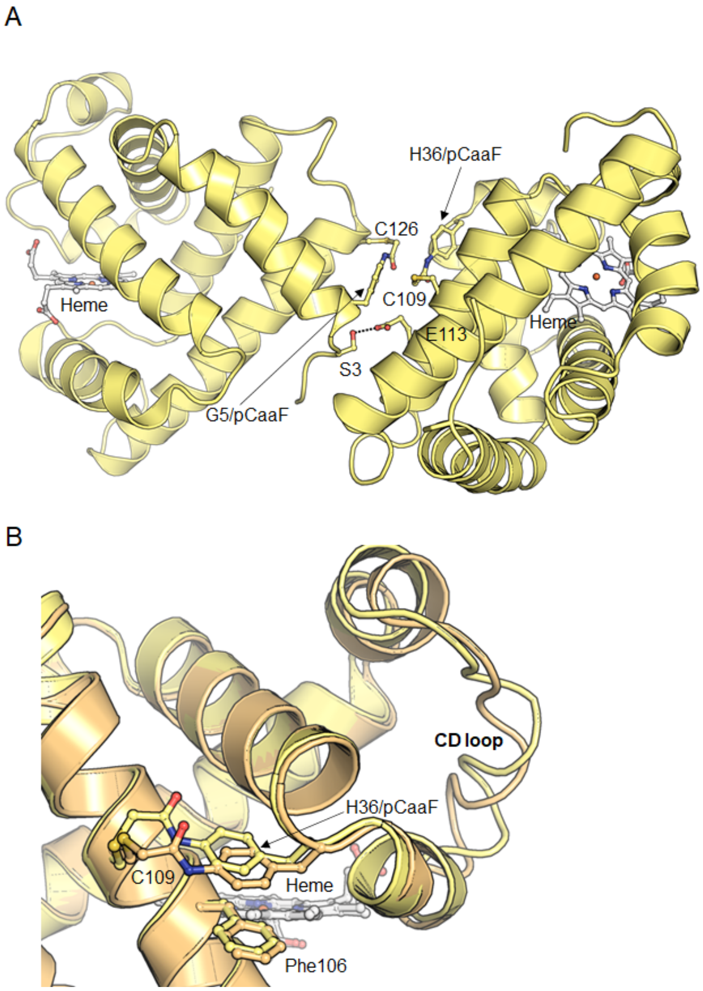

Figure S14. Comparison of crystal structures with design model. Design model (green CPK) shows high overall agreement with both sMb13($P2_12_12_1$)(yellow CPK, A and B) and sMb13($P2_1$)(purple CPK, C and D). Differences are observed presumably due to altered crystal packing in the experimentally determined structures. For sMb13(pCaaF) ($P2_12_12_1$) structure, there is a ~30° rotation in $\chi 2$ of the pCaaF compared to the design model (**A**) in the 5/126 staple, whereas the 36/109 staple is fully superimposable with the design model (**B**). In contrast, for the sMb13(pCaaF) ($P2_1$) structure, the 5-126 staple is nearly fully superimposable (**C**), whereas Cys109 adopts χ_2 values of -75.0° (g-) and 168.3° (t) in the crystal structure and design model, respectively (**D**). A small change in the χ_1 of pCaaF36 residue from -38.6° (crystal structure) to -46.6° (design model) is also observed.

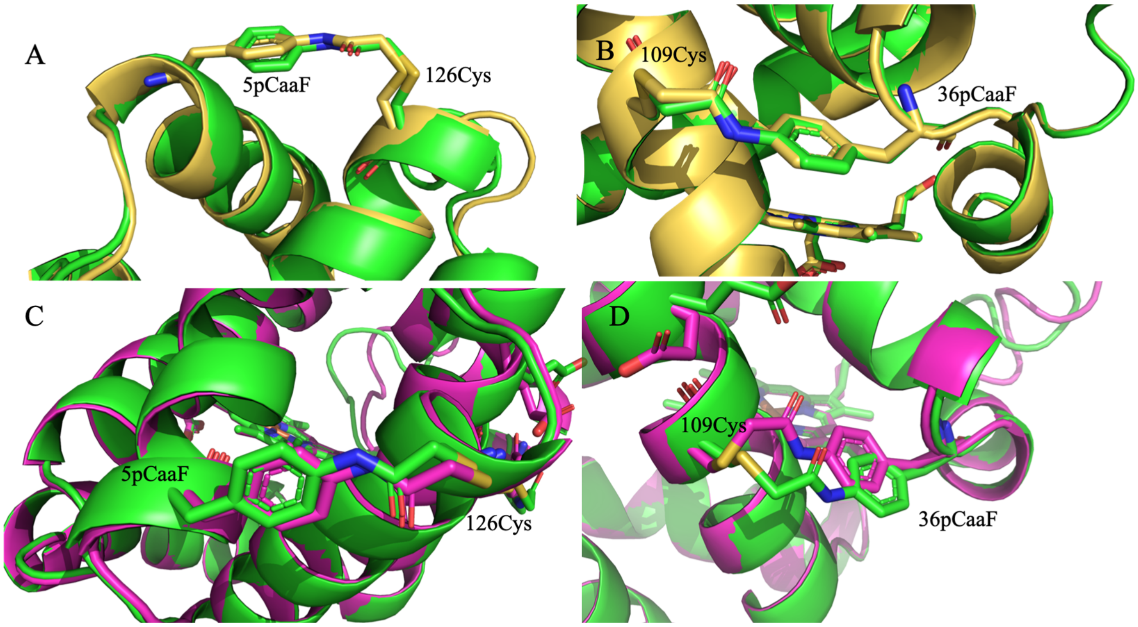

Figure S15. Cartoon putty representations and B-factors for structures (**A**) Mb(H64V,V68A), (**B**) sMb5(O2beY), (**C**) sMb5(pCaaF), (**D**) sMb13(pCaaF, $P2_1$), (**E**) sMb13(pCaaF, $P2_12_12_1$). Coloring and thickness of putty models are associated with B-factor values of 5-40 where blue is lowest B-factor and red is highest (white is intermediate). Asterisks mark regions of interest. **(F)** Residue-by-residue B-factor plot for structure main chain atoms. Residue-by-residue B-factor analysis was carried out as well as mapping of the B-factors onto the structures. This analysis revealed comparatively high B-factor values for N and C-termini, as expected, as well as three other regions with elevated B-factors, namely (i) the CD loop region and adjacent helices, which are in close proximity to the heme cofactor (**A, E**); (ii) the EF loop and N-terminus of the F helix (**B**); iii) the GH loop region from residues 119-123 (**C-E**). Notably, the parent protein and sMb13(pCaaF) ($P2_12_12_1$) structures show comparatively high values for region (i) that is in close proximity to the heme (**A, E**). Region (ii) shows relatively high B-factors for the sMb5(O2beY) variant (**B**), which may be stabilized through additional crystallographic symmetry contacts in the other crystal forms. For the pCaaF-containing variants, the relatively high B-factors for loop region (iii) (**C-E**) is observed in all three structures. Since the two sMb13(pCaaF) structures crystallized in different space groups, the higher B-factors for this region may not be due to crystal packing artefacts, but rather may be due to the fact that these three structures have in common the nearby 5(pCaaF)/126(Cys) pCaaF staple.

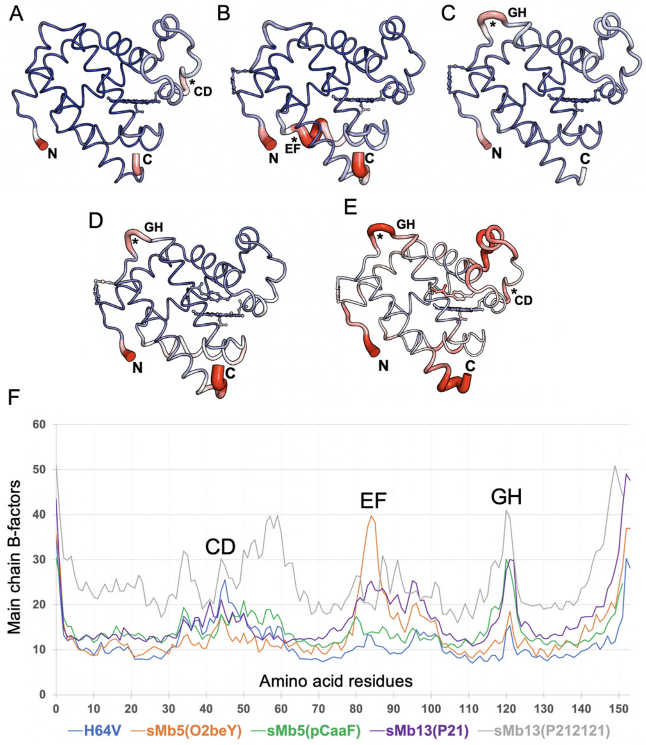

Crystallization experiments

Mb(H64V,V68A) was previously crystallized to 1.1-Å resolution (PDB: 6M8F) in spacegroup P6 using ammonium sulfate as the precipitant (Tinoco *et al.*, *ACS Catalysis,* 2019, *9*, 1514-1524). It is well established that sperm whale myoglobin protein has a proclivity for crystallization with ammonium sulfate as the precipitant. However, our initial attempts to crystallize the stapled variants under conditions similar to those used for the Mb(H64V,V68A) were unsuccessful at growing the P6 form of the protein crystals. We therefore screened the stapled variants using a 96-well sparse matrix screen that utilized ammonium sulfate as the main precipitant at various pH values, concentrations of ammonium sulfate and additives (Nextal DWBlock AmSO4 Suite). We were able to grow new crystal forms using this strategy, with the stapled variants forming crystals with different morphologies, both from each other and from the previously grown P6 crystals. We thus discovered new crystal forms for three of these stapled variants that differed from the P6 form. Both sMb5 variants sMb5(O2beY) and sMb5(pCaaF) crystallized in spacegroup $P2_12_12_1$, but the crystals were of different form and unit cell parameters, whereas the sMb13(pCaaF) variant crystallized in both a new spacegroup $P2_1$ and the same $P2_12_12_1$ form as sMb5(pCaaF). Structures of sMb5(O2beY), sMb5(pCaaF) and the double stapled sMb13(pCaaF) variants were determined to resolutions of 1.7, 1.17 and 1.3 Å resolution, respectively (**Table S2**).

Table S1 Rosetta-calculated energy units (REU) of stapled myoglobin variants. Constraint scores are a penalty score (the lower the better) which measure the degree of non-ideality of the cross-link.

**myoglobin variants**	**eUAA**	**total energy scores (REU)**	**constraint scores**
Mb(H64A,V68A)	none	-475.23	not applicable
sMb2	O2beY	-484.62	0.75
sMb2	pCaaF	-481.24	0.46
sMb2	pAaF	-484.08	0.72
sMb2	pVsaF	-499.43	0.73
sMb5	O2beY	-485.12	0.40
sMb5	pCaaF	-489.52	0.07
sMb5	pAaF	-489.58	0.21
sMb5	pVsaF	-494.61	0.79
sMb7	O2beY	-477.54	0.34
sMb7	pCaaF	*-466.31*	*4.45*

Table S2. Crystallographic data collection, processing, and refinement statistics for stapled Mb variant strucures.

	sMb5 (O2beY) 7SPE	sMb5 (pCaaF) 7SPF	sMb13 (pCaaF) 7SPG	sMb13 (pCaaF) 7SPH
**Data Collection**[a]				
Space group	$P2_12_12_1$	$P2_12_12_1$	$P2_12_12_1$	$P2_1$
Unit cell (Å)	a= 34.4, b = 54.3, c = 76.6<br>$\alpha = \beta = \gamma = 90.0$	a = 42.4, b = 56.0, c = 65.9<br>$\alpha = \beta = \gamma = 90$	a = 42.5, b = 56.5, c = 66.0<br>$\alpha = \beta = \gamma = 90$	a = 49.0, b = 41.6, c = 50.9<br>$\alpha = 90, \beta = 112.70, \gamma = 90$
Wavelength (Å)	1.5418	0.9795	0.9795	0.9795
Resolution range (Å)	30.00 – 1.70 (1.73 – 1.70)	35.65 – 1.17 (1.19 – 1.17)	35.73 – 1.30 (1.32 – 1.30)	31.15 – 1.30 (1.32 – 1.30)
Total observations	43788	654432	374839	283189
Total unique observations	14910 (430)	53004 (2284)	39628 (1961)	42991 (1314)
R_{merge}	0.120 (0.578)	0.047 (1.125)	0.051 (1.278)	0.039 (0.602)
R_{pim}	0.074 (0.542)	0.014 (0.396)	0.018 (0.427)	0.016 (0.281)
$\langle I/\sigma(I)\rangle$	10.5 (1.5)	25.3 (1.9)	17.0 (1.7)	22.3 (2.2)
$CC_{1/2}$	0.993 (0.634)	1.00 (0.673)	0.999 (0.715)	1.00 (0.818)
Completeness (%)	89.9 (53.0)	98.7 (86.6)	99.5 (99.7)	92.2 (57.9)
Multiplicity	2.9 (1.3)	12.3 (8.6)	9.5 (9.5)	6.6 (5.3)
Solvent content (%)	38.7	43.8	44.5	54.1
Wilson B	15.8	13.1	18.9	14.3
**Refinement Statistics**				
Resolution range (Å)	27.19 – 1.70	35.65 – 1.17	35.73 – 1.30	31.15 – 1.30
Reflections (total)	14797	52931	39563	42977
Reflections (test)	738	2607	1957	2157
Total atoms refined	1428	1419	1444	15033
R_{work}/R_{free}	0.18/0.21	0.15/0.17	0.16/0.18	0.13/0.16
RMSD bond lengths (Å)/ angles (°)	0.006/0.850	0.011/1.194	0.012/1.246	0.013/1.071
Ramachandran plot favored/allowed (%)	98.7/1.3	97.3/2.7	97.2/2.8	97.3/2.7
Mean B value for all atoms ($Å^2$)	16.3	18.0	28.8	21.3
Mean B value for protein atoms ($Å^2$)	15.2	16.2	27.5	18.6
Mean B value for solvent atoms ($Å^2$)	25.5	31.4	38.3	36.5
Mean B value for heme atoms ($Å^2$)	13.4	11.2	19.2	15.2
Molprobity all-atom clashscore	4.3	1.5	2.7	1.6

[a] Values in parentheses for data collection statistics refer to the high-resolution shell.

Table S3. RMSD values for aligned Mb variant structures.

	**sMb5 O2beY**	**sMb5 pCaaF**	**sMb13 $P2_12_12_1$**	**sMb13 $P2_1$**
**H64V**	0.258 (0.401)	0.315 (0.618)	0.269 (0.559)	0.368 (0.776)
**sMb5 O2BeY**		0.408 (0.664)	0.363 (0.617)	0.332 (0.815)
**sMb5 pCaaF**			0.170 (0.203)	0.472 (0.874)
**sMb13 $P2_12_12_1$**				0.470 (0.810)

Structures were aligned using Pymol "align" and main chain atoms (n+ca+c+o).
Values in parentheses are RMSD values for the aligned structures without removal of outliers.

Table S4. Table of crystallographic close contacts.

	0-2	A 3-18	19	B 20-35	C 36-42	43-50	D 51-57	E 58-76	77-82	F 83-95	96-99	G 100-118	119-123	H 124-149	150-153	Heme
**6M8F**	2	4, 15, 16, 18	19	21, 31		48, 50	51, 53, 54	66, 70	77, 80, 81	87, 91, 95	98	109, 112, 113, 116, 118	119, 120, 121, 122, 123	124, 125, 126, 128, 129, 132, 147, 148, 149	150, 151	No
**sMb5 O2beY**		3, 4, 5, 8, 11, 18	19	20, 21, 22, 27	36, 38, 41	48, 49, 50	51, 53, 54, 57	58, 59, 60, 62, 63, 66	77, 79	91, 95		102, 105, 106, 109, 112, 116, 117, 118	120	125, 126, 128, 132, 148, 149	150, 151	No
**sMb5 pCaaF**		5,8,12,15,16,18	19		38,41	44,45,47,48,50	51,53,54	67,71,74		83,84,85,87	91, 95, 96	100, 102,103	121, 122	124, 125, 126, 145, 148, 149		Yes
**sMb13 $P2_12_12_1$**		5,8,12,15,16,18	19		38,41	44,45,47,48,50	51,53,54, 59	67,71,74		83,84,85,87	91, 95, 96	100, 102,103	120, 121, 122	124, 125, 126, 145, 148, 149		Yes
**sMb13 $P2_1$**	1,2	3, 4, 5, 18	19	20, 21, 22, 31	36, 38, 41, 42	48, 49	51, 54, 57	58, 59, 62, 70	77	84		100, 103, 109, 113, 116		125, 128, 147, 148, 150	152	No

Intermolecular symmetry contacts were calculated using CCP4 Contact. Residues with contact distances between 0.0 and 4.0 Å are reported.
